# Cerebellum Transcriptome of Mice Bred for High Voluntary Activity Offers Insights into Locomotor Control and Reward-Dependent Behaviors

**DOI:** 10.1371/journal.pone.0167095

**Published:** 2016-11-28

**Authors:** Kelsey Caetano-Anollés, Justin S. Rhodes, Theodore Garland, Sam D. Perez, Alvaro G. Hernandez, Bruce R. Southey, Sandra L. Rodriguez-Zas

**Affiliations:** 1 Department of Animal Sciences, University of Illinois at Urbana-Champaign, Urbana, IL, United States of America; 2 Beckman Institute for Advanced Science and Technology, University of Illinois at Urbana-Champaign, Urbana, IL, United States of America; 3 Center for Nutrition, Learning and Memory, University of Illinois at Urbana-Champaign, Urbana, IL, 61801, United States of America; 4 Department of Biology, University of California Riverside, Riverside, CA, United States of America; 5 High-Throughput Sequencing and Genotyping Unit, Roy J. Carver Biotechnology Center, University of Illinois at Urbana-Champaign, Urbana, IL, United States of America; 6 Department of Statistics, University of Illinois at Urbana-Champaign, Urbana, IL, United States of America; 7 Carle Woese Institute for Genomic Biology, University of Illinois at Urbana-Champaign, Urbana, IL, United States of America; Universidade de Sao Paulo, BRAZIL

## Abstract

The role of the cerebellum in motivation and addictive behaviors is less understood than that in control and coordination of movements. High running can be a self-rewarding behavior exhibiting addictive properties. Changes in the cerebellum transcriptional networks of mice from a line selectively bred for High voluntary running (H) were profiled relative to an unselected Control (C) line. The environmental modulation of these changes was assessed both in activity environments corresponding to 7 days of Free (F) access to running wheel and to Blocked (B) access on day 7. Overall, 457 genes exhibited a significant (FDR-adjusted P-value < 0.05) genotype-by-environment interaction effect, indicating that activity genotype differences in gene expression depend on environmental access to running. Among these genes, network analysis highlighted 6 genes (Nrgn, Drd2, Rxrg, Gda, Adora2a, and Rab40b) connected by their products that displayed opposite expression patterns in the activity genotype contrast within the B and F environments. The comparison of network expression topologies suggests that selection for high voluntary running is linked to a predominant dysregulation of hub genes in the F environment that enables running whereas a dysregulation of ancillary genes is favored in the B environment that blocks running. Genes associated with locomotor regulation, signaling pathways, reward-processing, goal-focused, and reward-dependent behaviors exhibited significant genotype-by-environment interaction (e.g. Pak6, Adora2a, Drd2, and Arhgap8). Neuropeptide genes including Adcyap1, Cck, Sst, Vgf, Npy, Nts, Penk, and Tac2 and related receptor genes also exhibited significant genotype-by-environment interaction. The majority of the 183 differentially expressed genes between activity genotypes (e.g. Drd1) were under-expressed in C relative to H genotypes and were also under-expressed in B relative to F environments. Our findings indicate that the high voluntary running mouse line studied is a helpful model for understanding the molecular mechanisms in the cerebellum that influence locomotor control and reward-dependent behaviors.

## Introduction

The role of cerebellum in the control of movement has been extensively studied. However, the roles of the cerebellum in motivation, executive control, working memory, learning, and addictive behaviors are starting to be understood [1, 2].For example, the cerebellum has been associated with cocaine-related behaviors [3] as well as motor skills, object manipulation, knowledge, and their automatization [4]. Also, the cerebellum is activated by drug-associated cues [5–7] and during cognitive tasks such as language and memory in humans [8], and has been linked to reward-based learning [9, 10]. The involvement of the cerebellum in motivation or the internal drive of an organism may be established through its interactions with the endocrine system [11]. Indicators of exploratory behavior and spatial orientation in cerebellectomized rodents indicate that the cerebellum is involved not only in cognitive but also in motivational processes, spatial memory, and in cognitive processes of the motor program elaboration [12, 13].

Mouse lines selectively bred for high physical activity, such as the High Runner lines, are offering insights into the neurobiology of increased voluntary wheel running behavior [14–16]. Mouse lines selected for high voluntary wheel running exhibit significant behavioral and physiological differences relative to control lines as early as 10 generations after selective breeding. Moreover, studies of these lines are characterizing the role of brain regions in locomotor control [14, 15, 17–19]. Mice from the High Runner lines show significantly lower monoamine concentrations than mice from the control lines in the substantia nigra pars compacta and dorsolateral striatum regions of the brain, both of which are involved in locomotor control [20]. Also, blocked access to a wheel elicits neurobiological profiles similar to narcotic withdrawal in High Runner lines [16, 21].

Studies of high and low voluntary wheel-running rat and mouse lines have led to the proposition that this physical activity model can support the understanding of genes related to the motivation to run and to develop and maintain addictive behaviors in addition to locomotor activity [16, 22]. Physical activity and drugs of abuse have rewarding effects supported by similar brain pathways. High Runner lines also exhibit dysregulation in dopamine signaling [23] and endocannabinoid system involved in brain reward processes [16, 24]. High running can be a self-rewarding behavior exhibiting addictive properties [15, 17] and significant departures from average home cage activity levels have been associated with other behavioral disorders [25]. High Runner mouse lines also exhibit high home cage activity in the absence of wheels and high withdrawal behavior of despairity in a forced-swim test after removal of wheels following 6 days of access [26]. Likewise, when High Runner and control mice are rewarded with running time after pressing a lever [27], most High Runner and control mice press the lever when the reward was 30 minutes of running time but few High Runner mice press the lever when the reward was reduced to 1.5 minutes. The weaker response of the High Runner mice for limited reward suggests higher motivation for extended physical activity rather than potentially shorter attention because the latter would have been expressed at both reward levels.

Research on the High Runner mouse lines has concentrated on the striatum because of the association of this brain region with motor behavior [28–30], reward processing and addiction. However, changes in the cerebellum in response to selection are also expected because of the involvement of this brain region in the control of movement and motivational processes. Although the size of the cerebellum does not differ significantly between High Runner and control mice [15], selection may alter the molecular profiles and thus impact on behavior and locomotor control. Potential differences in the cerebellum transcriptome between mice from a high activity and unselected control lines and in consideration of environmental opportunities for activity have not been assessed.

A study of cerebelloctomized mice concluded that the cerebellum is involved in motor and spatial capabilities, and in cognitive and motivational processes that are key for exploration behavior [12]. Likewise, a study of the capability of cerebelloctomized rats to locate a reward concluded that the cerebellum is involved in the mechanisms sustaining focused spatial memory and in the cognitive processes of the motor program elaboration [13]. A similar association between the cerebellum and motor control, motivation, and reward-focus may have been developed in the High Runner mice line and a transcriptome analysis can offer insights into these roles.

The goals of this study were: 1) to compare the cerebellum transcriptome and associated networks of a High Runner mouse line relative to mice from a control line and, 2) to evaluate the modulation of potential transcriptome differences between genotypes and by the environmental opportunity for physical activity. We profiled the cerebellum transcriptome of mice using a 2-by-2 factorial design comparing 2 activity genotypes, a line selected for high wheel running versus an unselected control line, in either blocked or free access to wheel running environments. The comparison of both activity genotypes blocked and free access to wheel running environments permits us to disentangle the transcriptome profiles and networks related to the predisposition for high running as well as gene expression patterns correlated with actual running itself. Transcriptomic profiles in the brains of mice selectively bred for high voluntary wheel running in environments where the potential reward of wheel running is present or absent revealed molecular mechanisms underlying reward-dependent behaviors, motivation, and coordination of increased physical exercise. This study supports. efforts to understand the role of the cerebellum in cognition, motivation, and addictive behaviors.

## Materials and Methods

### Subjects

Mice from generation 66 of a replicated selective breeding experiment for high voluntary wheel-running behavior were used. As previously described [18], the original progenitors were outbred, genetically variable laboratory house mice (*Mus domesticus*) of the Hsd:ICR strain. After 2 generations of random mating, mice were randomly paired and assigned to 8 closed lines (10 pairs in each). In each subsequent generation, ~6 to 8 week-old offspring of these pairs were housed individually with access to a running wheel for 6 days (except during generations 32–35 when selection was relaxed as the colony was moved from Wisconsin to California). The highest-running male and female from each family within 4 High-Runner lines were selected as parents of the next generation. The selection criterion was total number of revolutions run on days 5 and 6 of the 6-day test. A male and a female were randomly chosen from within each family within 4 Control lines. Within all lines, the selected parents were randomly paired while avoiding sibling matings. In the present study, mice from 1 Control line (line 1) and 1 High-Runner line (line 7) chosen at random among all 8 lines available were evaluated in 2 activity environments. A total of 16, 7-week old adult males were used (n = 4 per line and environment combination). Power calculations based on gene expression measurements reported in prior studies [23, 31] involving these High-Runner lines indicated that a sample size of 4 provided 75% statistical power for a type I error rate corresponding to an FDR-adjusted P-value equal to 0.05 and log2(fold change) equal to 2.

Animal procedures were approved by the Illinois Institutional Animal Care and Use Committee and were in accordance with the National Institutes of Health Guide for the Care and Use of Laboratory Animals. As described previously [18], mice were weaned at 21 days of age, and individually marked. At approximately 6 weeks of age, mice were placed individually with access to wheels for 7 days. Mice were kept on a 12-hour light/dark cycle with lights on at 06:00 hours and lights off at 18:00 hours [32]. On day 7, half of the mice were blocked from entering the running wheel by placing a barrier in the tunnel connecting the wheel to the cage [21] During the mid-portion of the dark cycle, when mice are normally most active on running wheels, mice were removed from their cages and immediately decapitated. The Control and High Runner lines used in this study are denoted as activity genotypes C and H, respectively. The conditions blocked from running and free access to the wheels following a period of wheel availability are denoted environments B and F, respectively.

### RNA profiling

Brain was extracted and the whole cerebellum was dissected on a chilled aluminum block, snap frozen on dry ice and stored in a centrifuge tube at -80°C following published protocols [33]. Subsequently, tissue was homogenized with an RNase-free disposable pellet pestle (Fisher) and RNA was extracted using the commercially available RNeasy^®^ Lipid Tissue Mini Kit (Qiagen, Valencia, CA). Purification of the isolated RNA included treatment with DNase I (Qiagen, Valencia, CA), accordingly to the manufacturer's instructions. For assessing total RNA yield, aliquot samples were measured with the Qubit^®^ 2.0 (Life Technologies, Carlsbad, CA). Quality and integrity of isolated RNA samples were determined by 28S/18S rRNA analysis with the Agilent 2100 Bioanalyzer (Agilent Technologies, Santa Clara CA). RNA integrity was assessed using the Agilent 2100 Bioanalyzer with RNA Pico chip (Agilent Technologies, Palo Alto, CA). All samples had RNA Integrity Numbers (RIN) > 9. Libraries from individual mouse cerebellum samples were sequenced [34] and 100nt-long paired-end reads from each mouse separately were obtained using Illumina HiSeq 2000 (Illumina, San Diego, CA, USA).

### Differential expression analysis

Using the FastqGroomer tool, FastQ data files were converted to FastqSanger format following proven protocols [34, 35]. A quality control check was performed on the reads using FastQC, and the threshold for end position removal due to low quality using the Fastq Quality Trimmer tool was Phred < 20. The reads were then mapped to the mouse mm10 genome assembly (UCSC; http://genome.ucsc.edu) using Tophat (v2.0.13) [36]. Tophat settings were expected (mean) inner distance = 200nt, anchor length junctions spanned by reads with at least 8bp on each side of the junction with 0 mismatch, independent mapping of read segments of 25nt long, and default intron length specifications. Cufflinks (v2.2.0) [36] was used to assemble the transcripts and estimate gene abundances. Differential transcript abundance between experimental conditions was tested using Cuffdiff using a geometric library normalization [36]. Differential expression was tested between genotype-environment combination groups, between activity genotype groups, and between environment groups. Evaluation of the interaction between activity genotypes (H or C) and environments (B or F) allowed the identification of differential profiles between high activity and control genotypes that were environment-dependent [35].

The nomenclature used in this study to identify the genotype-environment combination uses 2 letters, the first letter specifies genotype and the second letter specifies environment. Four genotype-environment combination groups are available in this study: High runner genotype-Free environment (HF); High runner genotype-Blocked environment (HB); Control genotype-Runner environment (CF); and Control genotype-Blocked environment (CB). The 4 genotype-environment groups allowed the evaluation of 6 distinct pairwise contrasts: CB-HB, CB-CF, CB-HF, HB-CF, HB-HF, and CF-HF. While information on all these 6 contrasts is provided, the interpretation focuses on 3 orthogonal contrasts: CF-HF, CB-HB, and HB-HF. The first 2 orthogonal contrasts allowed the identification of differences between H and C activity genotypes within each environment and the third contrast allowed us to identify differences between B and F environments within the H genotype. Commonalities between the first 2 orthogonal contrasts support the identification of genes associated with activity genotype regardless of environment. Commonalities between all these orthogonal contrasts identify genes that may respond to both activity genotype and environment. Discussion based on these 3 orthogonal contrasts is complemented with information from selected other contrasts to facilitate the interpretation of results supporting the interaction between genotype and environment. Testing for the main effects of genotype and environment followed the testing for interaction. False discovery rate (FDR) adjusted P-values were used for multiple test adjustment of differential expression across all genes tested [37].

### Functional and network analysis

Enrichment of functional terms among the genes in 3 orthogonal interaction contrasts (CF-HF, CB-HB, and HB-HF), the activity genotype contrast, and the environment contrast were investigated. The comparison of the enriched functional terms would reveal shared and distinct molecular processes across contrasts that are not obvious from the analysis of individual genes. Two complementary functional enrichment analyses of the transcriptome profiles were undertaken. First, among genes differentially expressed (FDR-adjusted P-value < 0.05) enrichment of functional categories and pathways was performed using the Database for Analysis, Validation, and Integrated Discovery system (DAVID) [38]. Gene Ontology (GO) functional categories investigated included biological process (BP), molecular function (MF), and pathways defined by the Kyoto Encyclopedia of Genes and Genomes (KEGG). Enrichment of Functional Annotation Tool (FAT) categories was studied in recognition of the redundancy between GO terms. The FAT categories comprise informative BP and MF terms while filtering broad and less informative terms. EASE scores (modified Fisher Exact) were used to assess the statistical significance of the enrichment of the individual terms. Clustering of categories based on shared genes further minimized redundancy between categories. The statistical significance of these clusters was summarized using the enrichment score that corresponds to the geometric mean of the EASE scores of the categories in the cluster [39–42]. DAVID analysis was complemented with Gene Set Enrichment Analysis (GSEA) [43]. This analysis was performed on the gene expression profiles of all genes profiled to uncover enriched categories within the up- and down-regulated genes within orthogonal contrasts [44]. Maximum and minimum cutoffs for gene set size within up- and down-regulated lists were 1000 and 5 genes respectively. The statistical significance of the enrichment was assessed using 1000 permutations and FDR adjustment for multiple testing.

Gene networks were reconstructed within the Cytoscape environment [45] using the Bisogenet package [46]. Networks encompass genes exhibiting significant (FDR-adjusted P-value < 0.05) differential expression in at least one of the 3 orthogonal contrasts (CF-HF, CB-HB, HB-HF) to facilitate visual comparison [47]. Networks connecting at least 15 differentially expressed genes directly or indirectly through intermediate genes not differentially expressed were considered. The comparison of the expression profiles within networks across contrasts reveal shared and distinct co-regulation patterns and complement the information from functional analysis. Networks depict gene relationships based on protein-protein interactions annotated in BIOGRID, HPRD, DIP, BIND, INTACT, and MINT databases [40, 46, 48].

## Results and Discussion

### Summary of RNA-Seq measurements

The quality and quantity of the RNA sequence reads was evaluated across samples. The number of reads and quality scores along the reads were comparable across samples from all 4 activity genotype-environment groups. The average quality score Phred of the reads was 30 indicating a 99.9% base call accuracy [49]. The percentage of reads mapped to the mouse genome was approximately 99% corresponding to an average of 129,718,676.5 total reads mapped per sample. Differential expression between genotype and environment groups was tested on 9691 genes.

### Activity genotype-by-environment interaction effects on the transcriptome

The differential expression of individual genes uncovered in the present study supports accumulating evidence that the cerebellum is involved in motor control, regulation of neurotransmitter and hormone pathways, addictive behaviors [1], and neuropeptide and prohormone processes. Overall, 457 genes exhibited a significant (FDR-adjusted P-value < 0.05, comparable in this study to an unadjusted P-value < 0.001) genotype-by-environment interaction effect. A list of genes exhibiting the most significant (average log2(fold change) > |2|, FDR-adjusted P-value < 0.005) interaction effects is presented in Table 1. The complete list of genes significantly (FDR-adjusted P-value < 0.05) differentially expressed for the activity genotype-by-environment interaction effect is provided in **Table A** in S1 File. Annotation of many of these genes to signaling, sensory, and motor processes orchestrated in the cerebellum and involved in addictive behaviors [4] was noted.

**Table 1:** Genes exhibiting significant (FDR-adjusted P-value < 0.005, average contrast log2(fold change) > |2|) activity genotype-by-environment interaction.

Gene		Contrast Log2(Fold Change)^c^						FDR
Name^a^	UCSC ID^b^	CB-HB	CB-CF	CB -HF	HB-CF	HB-HF	CF-HF	P-value
Adora2a	uc007fqh.1	-4.02	-4.87	-4.72	-0.85	-0.70	0.15	2.57E-14
Arhgap8	uc007xch.2/uc007xck.2	2.07	-3.51	2.26	-5.58	0.19	5.76	2.31E-11
Cpne4	uc009rhr.1	-3.46	-4.77	-3.56	-1.31	-0.10	1.21	2.57E-14
Cpne5	uc008bsj.2/uc008bsk.2	-4.54	-5.76	-4.82	-1.22	-0.28	0.94	2.57E-14
Ctxn1	uc009ktp.2	-3.85	-4.60	-4.05	-0.75	-0.21	0.54	2.57E-14
Ddn	uc007xny.1	-5.12	-7.65	-6.92	-2.53	-1.80	0.73	2.57E-14
Drd2	uc009pja.1	-4.18	-4.84	-4.10	-0.66	0.08	0.74	2.57E-14
Egr3	uc007unb.1	-4.49	-5.93	-5.50	-1.44	-1.01	0.43	2.57E-14
Gda	uc008gyx.1	-4.61	-6.54	-5.30	-1.94	-0.69	1.25	2.57E-14
Gpr88	uc008rbz.2	-4.48	-6.43	-5.94	-1.94	-1.45	0.49	2.57E-14
Icam5	uc009oka.2	-4.58	-6.22	-5.70	-1.64	-1.12	0.52	2.57E-14
Kcnj4	uc007wtn.2	-4.60	-6.17	-5.48	-1.58	-0.88	0.69	2.57E-14
Lamp5	uc008mog.1	-2.96	-4.53	-3.88	-1.57	-0.92	0.66	2.57E-14
Lrrc10b	uc012bio.2	-4.44	-5.64	-5.26	-1.20	-0.82	0.38	2.57E-14
Nrgn	uc009ovc.1	-4.50	-5.51	-4.03	-1.01	0.48	1.48	2.57E-14
Nts	uc007gye.1	-3.36	-4.73	-3.36	-1.37	0.00	1.37	5.21E-08
Rxrg	uc007dla.2/uc007dlb.2	-3.84	-5.29	-4.37	-1.45	-0.52	0.92	2.57E-14
Scd4	uc008hpq.1	3.28	-1.66	3.17	-4.95	-0.11	4.84	2.57E-14
Sst	uc007ytx.1	-4.16	-4.61	-3.54	-0.45	0.62	1.07	2.57E-14
Tbr1	uc008jvd.1	-3.99	-4.94	-4.66	-0.95	-0.67	0.28	2.57E-14

^a^ Adora2a = adenosine A2a receptor; Arhgap8 = Rho GTPase activating protein 8; Cpne4 = copine IV; Cpne5 = copine V; Cyp11a1 = cytochrome P450, family 11, subfamily a, polypeptide 1; Ddn = dendrin; Drd2 = dopamine receptor D2; Gda = guanine deaminase; Gpr88 = G-protein coupled receptor 88; Icam5 = intercellular adhesion molecule 5, telencephalin; Kcnj4 = potassium inwardly-rectifying channel, subfamily J, member 4; Lamp5 = lysosomal-associated membrane protein family, member 5; Lrrc10b = leucine rich repeat containing 10B; Nrgn = neurogranin; Nts = neurotensin; Rxrg = retinoid X receptor gamma; Scd4 = stearoyl-coenzyme A desaturase 4; Sst = somatostatin; Tbr1 = T-box brain gene 1;

^b^ University of California Santa Cruz genome browser identifier.

^c^ Log2Fold is the log2-transformed fold change between 2 genotype-environment groups. When considering groups A vs B = A—B, Log2Fold Change = Log2 (A/B). Groups are: CB = low activity genotype-low activity environment; CF = low activity genotype-high activity environment; HB = High activity genotype-low activity environment; HF = High activity genotype-high activity environment

Among the genes exhibiting significant interaction, the number of significantly (FDR-adjusted P-value < 0.05) differentially expressed genes between specific contrasts were: 233 (CB-HB), 225 (CB-CF), 227 (CB-HF), 297 (HB-CF), 25 (HB-HF), and 248 (CF-HF). Transcriptome differences are more prevalent between activity genotypes (regardless of wheel-running availability in the environment), and between environments in the C genotype. Environmental differences were associated with substantially more differentially expressed genes (almost tenfold) in C genotype mice (CB-CF, 225 genes) than in H genotype mice (HB-HF, 25 genes). This result suggests that the environmental access to a running wheel, following previous opportunity to run on wheels, has limited impact on the transcriptome profiles beyond the impact of selection for high running at the genetic level. The environment that allows wheel running elicits differential gene expression in the C genotype that may already be activated in the H genotype.

Another finding from the evaluation of the 6 genotype-environment contrasts is that HB-CF exhibited the highest number of differentially expressed genes among all contrasts. This contrast compares two groups pairing “opposite” activity genotypes and environments (i.e. H genotype in B environment and C genotype in F environment). The abundance of differentially expressed genes in this contrast suggests that there may be synergistic effects between opposite levels of activity genotype and environment activity resulting in the highest number of gene differences. A possible hypothesis is that these transcript profiles support mechanisms enabling H genotype mice in the blocked-from-wheel running environment to experience exacerbated withdrawal behaviors whereas these mechanisms and associated withdrawal behaviors are not apparent in the C genotype mice in the F environment [16, 21].

Considering all genes differentially expressed at FDR-adjusted P-value < 0.05, the correlation between log2(CB-HB fold change) and log2(CB-CF fold change) is 0.77 (Table A in S1 File). In other words, the transcriptome comparison between control and high activity genotypes in this study within the B environment offers insights into the comparison between blocked and free activity environments within the C genotype. This finding supports our hypothesis that the physical activity genotypes studied are an effective model to understand the impact of availability of activity reward in the environment on the cerebellum transcriptome.

The contrast between environments within the H genotype (HB-HF) was selected a priori because this comparison would offer information on the lesser studied H genotype relative to the C genotype that is expected to resemble the baseline mouse population. A posteriori review of results indicated that environment differences elicited more extreme differential expression on the C relative to the H genotype among the genes exhibiting an interaction effect (Table 1 and Table A in S1 File). This result suggests that the environment reward conditions studied elicit comparable transcriptome responses in the H genotype whereas the cerebellum transcriptome of the C genotype is more susceptible to differences between the environments studied.

The profiles of the genes exhibiting significant genotype-by-environment interaction effect are consistent with the present understanding of the role of the cerebellum in locomotor regulation and the budding recognition of the role of cerebellum in reward-processing, goal focused- and reward-dependent behaviors. One common interaction profile among these genes was their simultaneous negative differential expression in the contrasts CB-HB, CB-CF, and CB-HF. This trend of under-expression in C relative to H genotype in the B environment, under-expression in the B relative to the F environment in the C genotype, and under-expression in the CB relative to the HF group was observed for P21 Protein Cdc42/Rac-Activated Kinase 6 (Pak6). This finding is consistent with a report of Pak6 deficiencies in mouse cerebellum associated with deficits in learning and locomotion [50]. The positive correlation between Pak6 abundance and activity genotype or environment detected in our study further validates the role of this gene on locomotor control, regardless of the genetic or environmental prompt.

Adenosine A2a receptor (Adora2a) had negative differential expression in the contrasts CB-HB, CB-CF, and CB-HF. This profile indicates that either H genotype or F environment alone or the combination of H genotype and F environment can trigger over-expression of this gene relative to the C genotype in the B environment. Consistent with our findings, studies have reported that adenosine receptor antagonists attenuate while adenosine uptake inhibitors augment ethanol-induced motor impairment in rodents via receptor populations in the cerebellum [51]. Adora2a is also involved in goal-focused behaviors and motor functions used to carry out those behaviors and in the absence of this receptor, mice display reduced exploratory behaviors and less motivation towards potentially addictive behaviors such as sexual activity and eating [52]. Moreover, Adora2a agonists significantly decrease cocaine induced hyperactivity in mice [53] and genetic variations on Adora2a affect psychomotor response speed and modulate the effect of the caffeine stimulant in humans [54]. The positive association between Adora2a mRNA abundance and the activity genotype motivated to run (H genotype) and environmental capability to run (F environment) uncovered in this study and with comparable phenotypes reported in previous studies reinforce the notion that this gene is a fundamental in the coordination of locomotion and reward-dependent behaviors.

Dopamine D2 receptor (Drd2) exhibited the same expression pattern as Adora2a. Drd2 had negative differential expression in the contrasts CB-HB, CB-CF, and CB-HF indicating that either H genotype or F environment alone or the combination of H genotype and F environment can trigger over-expression of this gene relative to the C genotype or B environment. The similarity between Drd2 and Adora2a expression patterns observed in this study supports prior implications of antagonistic activity between these genes. Drd2 knockout mice exhibit impaired locomotion and coordinated movements and treatment with Adora2a antagonists rescues the behavioral parameters [55]. Both the Drd2 knockout mice and the Adora2a antagonist treatment are extreme cases of the low Drd2 and Adora2a expression levels observed in the C genotype and B environment. The phenotypic associations observed on the former empirical studies support the differential transcriptional patterns between running genotype-environment groups uncovered in the present study.

The positive association between Drd2 level and motivation to run supports prior work on the effect of Drd2 in addiction and reward-dependent behaviors. Mice selectively bred for excessive exercise or obesity exhibit dopaminergic dysregulation [23]. Exercise elevates striatal dopamine D2 receptors in a mouse model of Parkinson’s disease [56] and dopamine receptors have been repeatedly detected in the cerebellar cortex [5]. Drd2 has been associated with addiction to opioids, nicotine, and cocaine [57] and mRNA encoding for Drd2 increase in response to cocaine treatment [58]. Studies of cerebellum in mice administered cocaine determined dysregulation in cerebral regions that have dopamine-signaling proteins [6, 59, 60]. In mice lacking D2 receptors, the rewarding properties of morphine were reduced [61]. The previous reports of positive associations between Drd2 levels and exercise and drugs of abuse are consistent with our findings of positive associations with high activity genotype and environment that may initiate, activate, and facilitate an inclination towards activity.

Copine 4 and 5 (Cpne4 and Cpne5, respectively) exhibited profiles similar to those observed in Adora2a and Drd2 (Table 1). Albeit less studied than Adora2a and Drd2, reports that genetic variants in Cpne5 were associated with alcohol dependence and obesity [62]. Cadherin 13 (Cdh13) exhibited a genotype-by-environment interaction effect and has been associated with d-amphetamine response [63] and with hyperactive or impulsive symptoms in attention deficit hyperactivity disorder cases [64]. The previous findings confirm the premise of a molecular link between high voluntary activity and goal- and reward-motivated behaviors through the cerebellum suggested in previous reports [22] and investigated in this study.

Another recurrent interaction profile displayed by transcripts such as Rho GTPase activating protein 8 (Arhgap8) and Stearoyl-coenzyme A desaturase 4 (Scd4) encompassed simultaneous gene over-expression in the CB-HB and CF-HF contrasts. Rho GTPase signaling is impaired in the cerebellum of a mice model of fetal alcohol syndrome [65] and several RhoGAPs are coded by the white rabbit gene in Drosophila, a gene that plays a key role in the regulation of both stimulant and sedative behaviors such as motor incoordination induced by ethanol [66]. Moreover, a study of the brain from mice with exercise-induced fatigue uncovered differential expression of Scd4 between fatigued and control mice [67]. The previous trends are consistent with our observations that Arhgap8 and Scd4 were over-expressed in mice from the C genotype compared to the H genotype in both activity environments.

Cytochrome P450, family 11, subfamily a, polypeptide 1 (Cyp11a1) was also over-expressed in the C genotype compared to the H genotype in both environments. The cerebellum hosts enzymatic process that allow neurosteroid synthesis and within this process the cholesterol side chain cleavage enzyme Cyp11a1 enables the production of pregnenolone. Alterations of the neurosteroidogenesis-related proteins are speculated to participate in dysregulated locomotor coordination involving cerebellar functions [68]. Also related to hormone processes, Peptidyl arginine deiminase, type IV (Padi4) exhibited the same pattern as Arhgap8 and Cyp11a1. Padi4 plays a role in hormone-induced transcription [69] and is a maker of oxidative stress, a process associated with drug addiction and psychiatric disorders [70, 71].

The expression of various neuropeptide prohormones and related receptor genes exhibited significant genotype-by-environment interaction. Prohormones included: adenylate cyclase activating polypeptide 1 (Adcyap1), cholecystokinin (Cck), somatostatin (Sst), VGF nerve growth factor inducible (Vgf), neuropeptide Y (Npy), neurotensin (Nts), proenkephalin (Penk), and tachykinin 2 (Tac2). The profile of these genes was characterized by significant under-expression in the CB group relative to the HB, CF and HF groups-HB and CB-CF contrasts. This profile suggests that the H genotype and F environment, individually or in combination, result in an elevation of the expression of these neuropeptide genes relative to the combination of baseline activity genotype and environment. Also, for neuropeptide genes, selection-control line differences in a non-reward environment elicited a transcriptome response similar to reward availability among unselected mice. This comparable impact of H genotype and F environment supports the paradigm that the running genotypes studied can serve as model for running availability environment and associated addiction and reward-dependent behaviors.

The gene expression trends observed in this study were consistent with previous studies of other strains of mice that considered neuropeptides in the cerebellum and addiction processes. The expression in the cerebellum of neuropeptides encoded in prohormone genes such as Penk, Npy, Nts, Sst, and Cck is well-reported [72] and associations with reward-dependent behaviors has been reported. Penk knock-out mice exhibit lower cannabinoid withdrawal syndrome than the corresponding wild type mice [73]. Npy, a peptide widely expressed in the cerebellum, modulates many functions and behaviors including anxiety that is associated with reward withdrawal conditions [74]. Moreover, the expression of Npy in the hypothalamus has been correlated with increased wheel running in rats [75]. A study of mice deficient in Sst demonstrated motor learning impairment, a function controlled by the cerebellum [76].

Transcripts of neuropeptide receptors, such as glucagon receptor (Gcgr), vasoactive intestinal peptide receptors 1 and 2 (Vipr1 and Vipr2), corticotropin releasing hormone receptor 2 (Crhr2), Npy receptor Npy1r, and opioid receptor delta 1 (Oprd1) also exhibited significant genotype-by-environment interaction effects and patterns similar to those detected in neuropeptide genes. Oprd1 modulates substance dependence risk [77]. Cerebellar astrocytes secrete neuroactive and vasoactive peptides including Npy in response to oxidative stress conditions [78]. Npy receptor knockout mice exhibited faster disappearance and reappearance of cocaine-induced conditioned place-preference and these receptors are expressed in the cerebellum [79]. Glucagon, vasoactive intestinal peptide, and secretin belong to the same family of bioactive peptides that have high homology and secretin receptors are abundant in the cerebellum [80]. Hemogloblin (Hba) A and B also exhibited significant genotype-by-environment interaction and prior studies have reported hemoglobin in mouse cerebellum [81]This result may imply the presence of peptides derived from hemoglobin, such as hemopressin, that have an important role in binding cannabinoid receptors [82–84]. These genes were over-expressed CF-HF indicating that selection has resulted in lower levels of hemogloblin gene expression in an environment where the activity reward is available.

**Fig 1:**
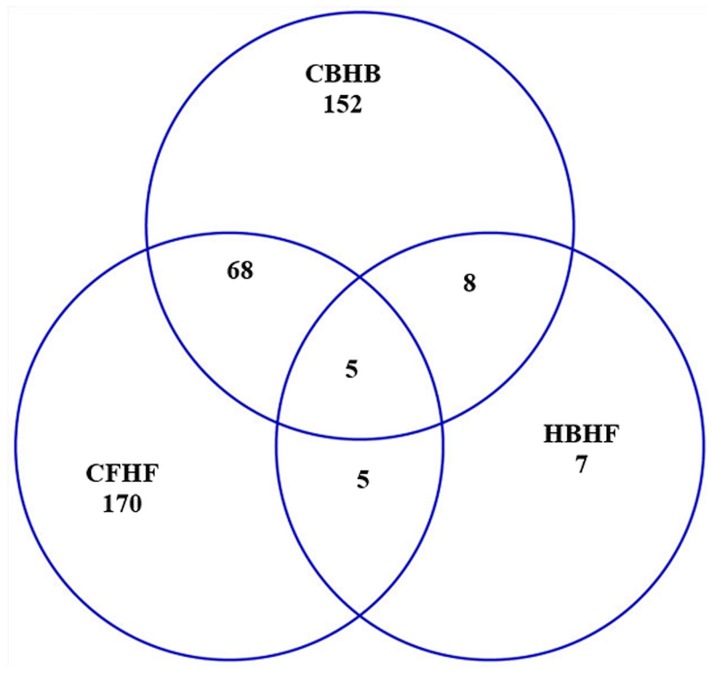
Number of significantly differentially (FDR-adjusted P-value < 0.05) expressed genes that overlap between low activity control genotype-high activity environment and high activity genotype- high activity environment (CF-HF), low activity control genotype-low activity environment and high activity genotype- low activity environment (CB-HB), and high activity genotype-low activity environment and high activity genotype- high activity environment (HB-HF).

Fig 1 presents a Venn diagram illustrating the differentially (FDR-adjusted P-value < 0.05) expressed genes shared between CF-HF, CB-HB, and HB-HF contrasts. The limited overlap between contrasts depicted in this diagram confirms that many of the differences in gene expression between activity genotypes are dependent on the environment. While 5 genes were differentially expressed in all 3 contrasts, 68 genes were differentially expressed in both activity genotype contrasts (CB-HB and CF-HF). This overlap offers insights into molecular mechanisms affected by activity genotype that are shared across activity availability environments. Among the genes differentially expressed between activity genotypes in both environments, Neurogranin (Nrgn) and Dendrin (Ddn) appear in the list of genes exhibiting the most significant interaction effect (FDR-adjusted P-value < 0.005, average log2(fold change) > |2|) presented in Table 1. Both Nrgn and Ddn displayed the previously described frequent interaction profile including negative differential expression in the contrasts CB-HB and CB-CF.

The Ddn profile observed in this study is consistent with reports that this gene is over-expressed in response to 3,4-Methylenedioxymethamphetamine (‘ecstasy’) in the dorsal raphe nucleus of mice [85]. The neuropeptide-like peptide Nrgn is expressed in murine Golgi cells that are GABAergic interneurons [86] and ablation of these Golgi cells results in severe motor disorders and inability to perform compound movements [87]. Nrgn is also associated with alcohol addiction through the role in synaptic plasticity and signal transduction processes [88]. The under-expression of Nrgn in the C genotype and B environment is consistent with limited motor coordination and movements observed in mice without Nrgn-rich Golgi cells previously reported. This finding, together with the association between addiction behaviors and Nrgn and Dnd, support the hypothesis that the genotypes and environments compared in this study offer insights into transcriptome underlying locomotor and reward-dependent phenotypes.

**Fig 2:**
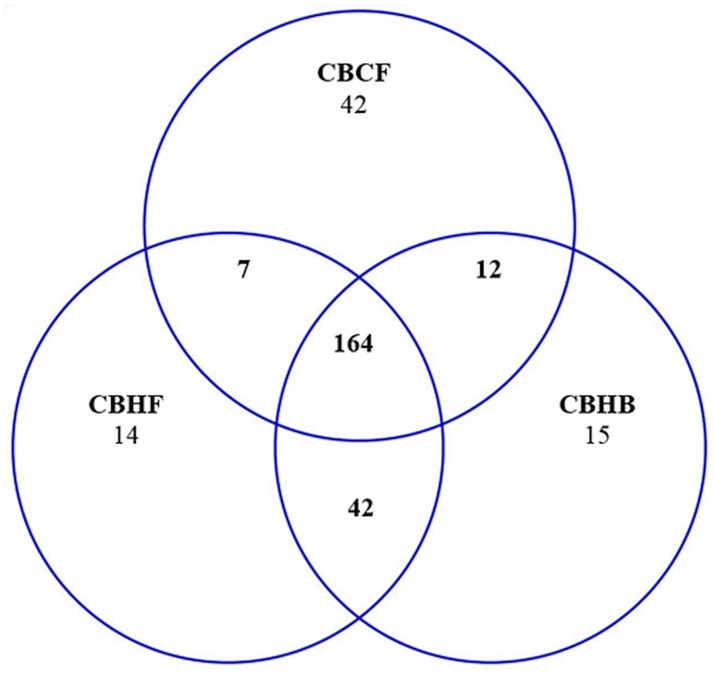
Number of significantly differentially (FDR-adjusted P-value < 0.05) expressed genes that overlap between low activity control genotype-low activity environment and high activity genotype- low activity environment (CB-HB), low activity control genotype-low activity environment and low activity control genotype- high activity environment (CB-CF), and low activity control genotype-low activity environment and high activity genotype- high activity environment (CB-HF).

The consideration of the 3 genotype-by-environment pairwise contrasts that include the baseline CB group offers a complementary view of the impact of activity interaction effects. Fig 2 illustrates the overlap in differentially (FDR-adjusted P-value < 0.05) expressed genes between all 3 contrasts that include the CB baseline group. Overall, 164 differentially expressed genes were shared between all 3 contrasts involving the genotype-environment combination CB, suggesting that either H genotype, F environment, or the combination of both conditions can elicit similar transcriptome changes relative to the CB baseline. Furthermore, this complementary perspective confirms that genotypic make-up leading to high voluntary activity is associated with changes in gene expression comparable to environmental wheel running restriction following a period of availability.

### Functional categories enriched among genes exhibiting activity genotype-by-environment interaction effects

The functional categories enriched among the genes exhibiting significant genotype-by environment interaction included processes associated with motor control and reward-dependent behaviors. This finding confirms the role of the cerebellum in locomotor regulation and offers evidence that the cerebellum also plays a role in molecular signaling connected with addictive behaviors [1]. Table 2 lists selected categories and functional clusters that were enriched among the genes exhibiting significant interaction (FDR-adjusted P-value < 0.05) by orthogonal contrast (CB-HB, CF-HF, and HB-HF) and that have an enrichment score > 1.5. A complete list of terms and enriched clusters is presented in **Table B** in S1 File.

**Table 2:** Functional clusters that have enrichment score > 1.5 and corresponding Gene Ontology (GO) biological process (BP), molecular function (MF) Functional Annotation Tool (FAT) categories, and KEGG pathways among the genes that exhibited significant genotype-by-environment interaction (FDR-adjusted P-value < 0.05) and differentially expressed (FDR-adjusted P-value < 0.05) in the 3 orthogonal genotype-by-environment contrasts (CB-HB, CF-HF, and HB-HF).

Contrast and Category	Term	Count	P-value	FDR P-value
CB-HB				
Cluster 1	Enrichment Score: 3.16			
GO BP FAT	GO:0030814~regulation of cAMP metabolic process	8	4.77E-06	0.01
GO BP FAT	GO:0007626~locomotory behavior	11	1.53E-04	0.24
GO BP FAT	GO:0050905~neuromuscular process	4	2.26E-02	1.00
Cluster 2	Enrichment Score: 2.62			
KEGG	mmu04080:Neuroactive ligand-receptor interaction	15	3.60E-07	0.0003
GO BP FAT	GO:0007186~G-protein coupled receptor protein signaling pathway	25	1.29E-01	1.00
Cluster 3	Enrichment Score: 2.03			
GO BP FAT	GO:0051588~regulation of neurotransmitter transport	5	6.28E-05	0.10
KEGG	mmu04020:Calcium signaling pathway	9	8.44E-04	0.90
GO BP FAT	GO:0031644~regulation of neurological system process	7	9.84E-04	1.00
GO BP FAT	GO:0042493~response to drug	3	2.22E-01	1.00
Cluster 4	Enrichment Score: 1.99			
GO MF FAT	GO:0042165~neurotransmitter binding	6	2.17E-03	1.00
GO MF FAT	GO:0042923~neuropeptide binding	4	7.23E-03	1.00
Cluster 5	Enrichment Score: 1.66			
GO BP FAT	GO:0007610~behavior	16	1.42E-05	0.02
Cluster 6	Enrichment Score: 1.58			
GO BP FAT	GO:0007631~feeding behavior	5	1.71E-03	1.00
CF-HF				
Cluster 1	Enrichment Score: 1.86			
GO BP FAT	GO:0015669~gas transport	4	3.21E-04	0.51
GO MF FAT	GO:0005344~oxygen transporter activity	3	5.85E-03	1.00

Central themes among the genes differentially expressed in the CB-HB contrast are the enrichment of regulation of locomotor behavior, neurotransmitter and neuropeptide binding processes, cyclic adenosine monophosphate (cAMP) metabolic process, neuro-related process, and response to drug. Locomotor behavior and neurotransmitter and neuropeptide categories are consistent with our previous discussion at the individual gene level. Enrichment of cAMP is consistent with reports that dopamine 2-like receptors inhibit adenylate cyclase and thus reduce the concentration of cAMP whereas dopamine 1-like receptors have the opposite effect [89]. The results from the functional analysis of the CB-HB contrast are consistent with studies reporting association between physical activity and neurological system processes [90, 91]. Among the genes that exhibited a significant interaction effect, the high correlation of log2(fold change) between CB-HB and CB-CF and comparable enriched categories confirm that the processes impacted by the selection for high physical activity are similarly impacted by the availability of activity reward in the environment. Enrichment of neuropeptide signaling processes encompassed mostly under-expressed neuropeptide genes including Adcyap1, Oprd1, melanin-concentrating hormone receptor 1(Mchr1), Npy, Npy1r and Penk and over-expressed genes Npy2r and Tac2.

Weaker enrichment trends were observed among the genes differentially expressed in the CF-HF contrast meanwhile the limited number of differentially expressed genes prevented significant enrichment in the HB-HF contrast (Table 2). The enrichment oxygen transport processes with a predominance of genes under-expressed in the CF-HF contrast may be associated with regulation of locomotion. The functional analyses offer a glimpse of how genetic selection for high voluntary running has altered the cerebellum molecular processes and may be behind the behavioral inclination, motivation, or addiction towards high physical activity as well as impacting their physical characteristics [15].

### Network of genes exhibiting activity genotype-by-environment interaction effects

**Fig 3:**
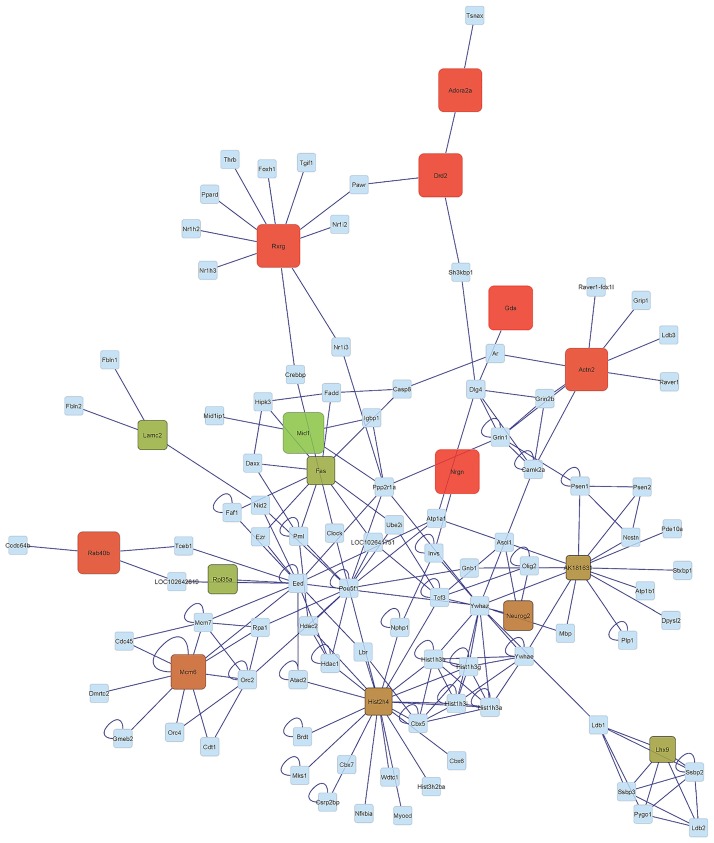
Network of genes significantly differentially expressed (FDR-adjusted P-value < 0.05) in the Control genotype-Blocked environment and High activity genotype-Blocked environment (CB-HB) orthogonal contrast. (Node Color: Red indicates under-expression, Green indicates over-expression, Gray indicates genes connecting differentially expressed gene but not differentially expressed in this study; Node Size: larger sizes indicates relatively higher fold changes, while smaller sizes indicates relatively smaller fold changes).

**Fig 4:**
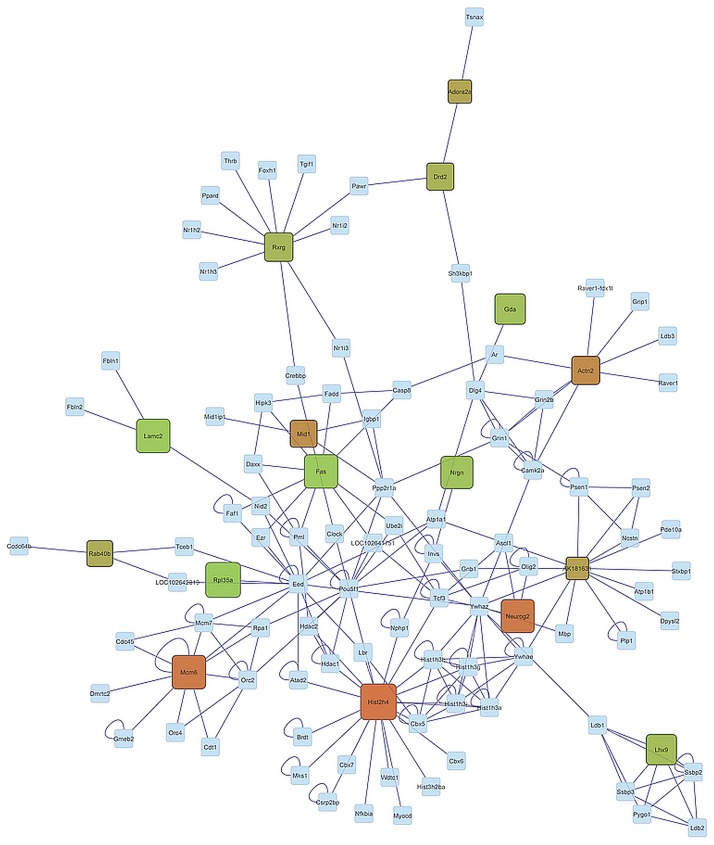
Gene network of genes significantly differentially expressed (FDR-adjusted P-value < 0.05) in the Control genotype-Free activity environment and High activity genotype-Free environment (CF-HF) orthogonal contrast. (Node Color: Red indicates under-expression, Green indicates over-expression, Gray indicates genes connecting differentially expressed gene but not differentially expressed in this study; Node Size: larger sizes indicates relatively higher fold changes, while smaller sizes indicates relatively smaller fold changes).

**Fig 5:**
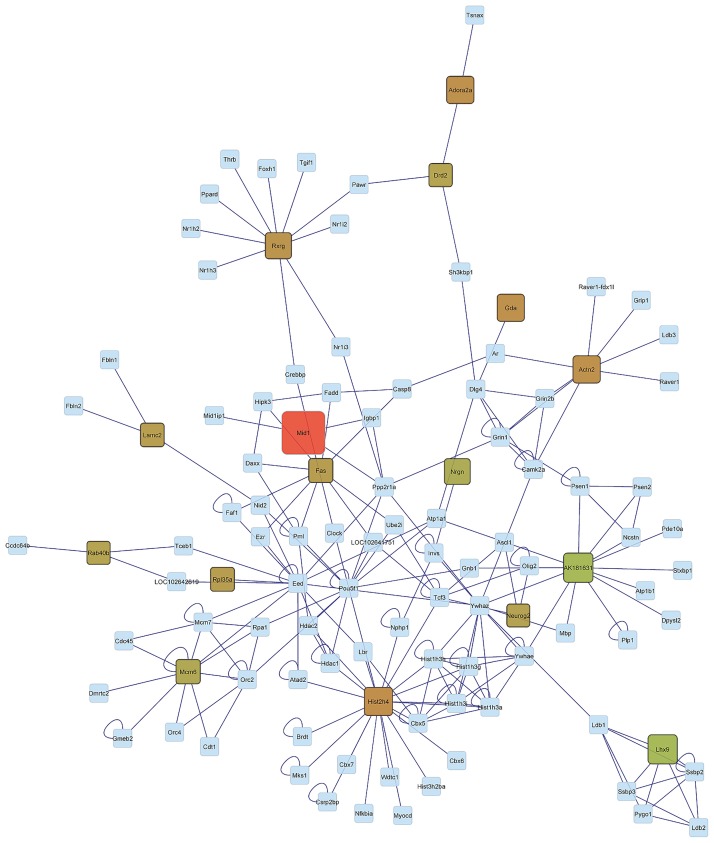
Gene network of genes significantly differentially expressed (FDR-adjusted P-value < 0.05) in the High activity genotype-Blocked environment and High activity genotype-Free environment (HB-HF) orthogonal contrast. (Node Color: Red indicates under-expression, Green indicates over-expression, Gray indicates genes connecting differentially expressed gene but not differentially expressed in this study; Node Size: larger sizes indicates relatively higher fold changes, while smaller sizes indicates relatively smaller fold changes).

Our previous findings offer insights into the expression profile of genes both individually and in functional clusters. Network analysis aids in understanding the relationship between genes and in assessing the connectivity among genes. The relationship among genes exhibiting significant (FDR-adjusted P-value < 0.05) activity genotype-by-environment interaction was depicted using networks. This visualization contributed to our understanding of the similarities in gene co-regulation between activity genotypes within the B and F environments (CB-HB and CF-HF in Figs 3 and 4, respectively) and between activity environments in the H genotype (HB-HF in Fig 5). Rectangular nodes represent the differentially expressed genes and edges represent the known associations between genes based on curated databases of molecular interactions.

The distribution of profiles in the networks CF-HF (comparing genotypes within the F environment) and CB-HB (comparing genotypes within the B genotype) appear more extreme meanwhile the distribution of profiles in the network HB-HF (comparing environments within the H genotype) appears intermediate. Meanwhile a number of genes are under-expressed in the CB-HB network, many genes are over-expressed in the CF-HF network. Also, the differential expression level appears more consistent across the CF-HF network than in the CB-HB network. These topologies confirm the differential response of the cerebellum transcriptome from the H genotype relative to the C genotype between activity environments.

The distribution of high differentially expressed genes between genotypes in the periphery of the network in the B environment (Fig 3) and in the center of the network in the H environment (Fig 4) offered insights into the interaction between genotype and environment. This comparison of topologies suggests that selection for high voluntary running is linked to a predominant dysregulation of hub genes in an environment that enables running whereas a dysregulation of ancillary genes is favored in an environment that blocks running. The premise that core components of a network are usually conserved and central in terms of functions, while periphery components are usually more variable and specialized [92] may offer insights into our finding that core genes tended to be dysregulated in the F environment while periphery genes tended to be dysregulated in the B environment. Based on this premise we postulate that expression changes in highly connected genes in the cerebellum of H genotype mice may be associated with the capacity of these mice to act upon their motivation to run and to execute high wheel running in the F environment

The evaluation of network components indicates that Laminin, Gamma 2 (Lamc2), Fas Cell Surface Death Receptor (Fas), and Ribosomal Protein L35a (Rpl35a) exhibited a similar differential pattern in the genotype contrast CB-HB and CF-HF. These genes were significantly over-expressed in mice from the H relative to the C genotype in both activity environments. The association of these genes with genotype activity dependence, irrespectively of reward availability in the environment suggests a fundamental disruption in the expression level that the environment is not able to offset. These genes have been associated with addictive behaviors. Lamc2 is down-regulated by acute nicotine exposure [93], Fas receptor proteins are modulated during opiate addiction in the rat brain [94], and Rpl35a was dysregulated in cell lines treated with nicotine [95]. LIM Homeobox 9 (Lhx9) was also over-expressed in the CF-HF contrast. Lxhr9 regulates the neuropeptide orexin that is involved in arousal, appetite, wakefulness, and reward processes [96]. The involvement of orexin in arousal wakefulness and reward systems [97] makes this gene a good candidate to understand the molecular mechanisms underlying the motivation to run.

Further consideration of the network components uncovers 6 genes including Nrgn, Drd2, Retinoid X Receptor Gamma (Rxrg), Guanine Deaminase (Gda), Adora2a, and Ras-Related Protein Rab-40B (Rab40b) that display opposite patterns between the CB-HB and CF-HF gene networks. This pattern indicates that environmental activity availability impacted the change in expression level between C and H genotypes. These genes are over-expressed in CF-HF and under-expressed in CB-HB. These trends confirm that genotype differences must be evaluated in the environmental context and this context-dependency may be applicable to other addictive behaviors.

Among the genes that exhibited distinct patterns between the CB-HB and CF-HF networks, Gda and Rxrg were under-expressed in C relative to H genotype mice in the B environment but over-expressed in the H environment. Under the premise that the comparison of H and C genotypes can help us understand reward dependent behaviors, the differential expression of Gda between genotypes in the B environment is consistent with a report of Gdaunder-expression in the brain of monkeys that self-administered cocaine [98]. Likewise, the differential expression of Rxrg between genotypes in the B environment is consistent with reports that over-expression of Rxrg in humans is associated with sensation seeking [99] and that Rxrg knock-out mice show impaired locomotion along with reduced dopamine signaling [100]. Also, ablation/loss of Rxrg signaling in mice leads to depressive behaviors due to the decreased dopamine signaling in the striatum, supporting the association of this gene with reward and pleasure [101]. In agreement with dopamine receptor trends previously described, Rxrg is a transcription factor that regulates striatal D1r gene expression [102] and retinoid receptors directly up-regulate Drd2 [103]. The present study uncovered several genes that exhibited environment-dependent differential expression between genotypes. This finding demonstrates the need to account for reward availability context or environments in studies of molecular mechanisms associated with high voluntary running. This finding is likely applicable to other genes and reward-dependent behaviors.

Most of the genes over- or under- expressed in the networks of activity genotype contrasts (CB-HB and CF-HF) are only intermediately differentially expressed between activity environments in the H genotype (HB-HF). This result is consistent with the overlap in differentially expressed genes between the 3 orthogonal contrasts presented in Fig 1. This result suggests that environment is not a major factor confounding the results of our genotype comparisons. The only exception is Midline 1 (Mid1). This gene was significantly over-expressed in the CB-HB contrast yet was significantly under-expressed in the HB-HF. In fact, Mid1 was the only significantly under-expressed gene in this network. This result could be linked to reports that Mid 1 is repressed in daDREAM mice that also exhibit significant suppression of neuromotor development early after birth [104]. Our finding suggests that a reward-available environment can counteract the dysregulation in Mid1 expression associated with high voluntary activity genotype.

### Effect of high voluntary activity genotype on the cerebellum transcriptome

In addition to environmental-dependent, differential expression patterns between activity genotypes that are environmental-independent were uncovered. Table 3 summarizes genes differentially expressed (log2(fold change) > |3| and FDR-adjusted P-value < 0.007) between low and high activity genotypes. A list of genes significantly differentially expressed at FDR-adjusted P-value < 0.05 is provided in **Table C** in S1 File. The majority of the 183 differentially expressed genes were under-expressed in the C relative to the H genotype mice. Selected genes that exhibited differential expression between the genotype groups and that can offer insights into high activity genotype differences in the cerebellum transcriptome are discussed.

**Table 3:** Genes significantly differentially expressed (FDR-adjusted P-value < 0.007 and log2(fold change) > |3|) between mice from Control (C) and High (H) activity genotypes.

Gene Symbol	Gene Name	UCSC ID^a^	Log2 (C/H)	FDR P-value
Foxg1	forkhead box G1	uc007nmf.2/uc011ylr.1	-5.20	0.006
Kcnv1	potassium channel, subfamily V, member 1	uc007vql.2/uc007vqm.2	-4.89	0.006
Sp9	trans-acting transcription factor 9	uc008kcj.2	-4.81	0.006
Dlx1	distal-less homeobox 1	uc008kau.1/uc008kav.1	-4.80	0.006
Gpr6	G protein-coupled receptor 6	uc007exj.1	-4.71	0.006
Drd1	dopamine receptor D1	uc011yzm.2	-4.58	0.006
Zfp831	zinc finger protein 831	uc008ofh.1	-4.48	0.006
Cpne5	copine V	uc008bsj.2/uc008bsk.2	-4.47	0.006
Kcnj4	potassium inwardly-rectifying channel, subfamily J, member 4	uc007wtn.2	-4.42	0.006
Ankrd63	ankyrin repeat domain 63	uc008lsh.1	-4.40	0.006
Gda	guanine deaminase	uc008gyx.1	-4.30	0.006
Lrrc10b	leucine rich repeat containing 10B	uc012bio.2	-4.28	0.006
Kcnf1	potassium voltage-gated channel, subfamily F, member 1	uc007ncn.2	-4.27	0.006
Chrm1	cholinergic receptor, muscarinic 1, CNS	uc008gmf.2/uc008gmg.2	-4.20	0.006
Kcng1	potassium voltage-gated channel, subfamily G, member 1	uc008oat.1	-4.01	0.006
Dlx2	distal-less homeobox 2	uc008kax.2	-3.94	0.006
Rxrg	retinoid X receptor gamma	uc007dla.2/uc007dlb.2	-3.80	0.006
Adora2a	adenosine A2a receptor	uc007fqh.1	-3.76	0.006
Tbr1	T-box brain gene 1	uc008jvd.1	-3.72	0.006
Rprml	reprimo-like	uc007lvn.1	-3.60	0.006
Crhbp	corticotropin releasing hormone binding protein	uc007rmk.2	-3.56	0.006
Rtn4rl2	reticulon 4 receptor-like 2	uc008kjm.1	-3.46	0.006
Vdr	vitamin D receptor	uc007xlk.1	-3.41	0.006
Actn2	actinin alpha 2	uc007pli.1/uc007plj.1	-3.34	0.006
Ptprv	protein tyrosine phosphatase, receptor type, V	uc007csr.2	-3.32	0.006
Kcnh4	potassium voltage-gated channel, subfamily H (eag-related), member 4	uc007lmf.2	-3.18	0.006
Neurl1b	neuralized E3 ubiquitin protein ligase 1B	uc008bed.2	-3.17	0.006
Sst	somatostatin	uc007ytx.1	-3.11	0.006
D430019H16Rik	RIKEN cDNA D430019H16 gene	uc007oyk.2	-3.06	0.006
Prss12	protease, serine 12 neurotrypsin (motopsin)	uc008rfl.2	-3.04	0.006

^a^ University of California Santa Cruz genome browser identifier.

Several genes differentially expressed between the C and H genotype mice have been associated with locomotor control, reward-dependent behaviors, neurological systems and signaling pathways. Dopamine receptor D1 (Drd1) was over-expressed in the cerebellum of H relative to C genotype mice. This result is consistent with the over-expression of Drd1 in the caudate-putamen of cocaine-sensitized mice relative to control [105] and reports that dopamine receptors D1 and D2 systems mediate the effects of cocaine on cerebellar neurons of adult rats [106]. Also, endogenous drug administration simulates D1 receptor, which leads to the expression of D2 receptor-mediated reward-driven behaviors and gene expression changes in non-cerebellar brain regions [105, 107–110]. Both D1-like and D2-like dopamine receptors are involved in the response of rats who are taught to perform a behavior in order to receive pleasurable electrical stimulation [111, 112]. All together, these results offer evidence of the involvement of Drd1 expression on reward-dependent behaviors and confirm that the High Runner line studied here is helpful to understand the transcriptome associated with these behaviors.

Muscarinic Acetylcholine Receptor M1 (Chrm1) was also over-expressed in mice from the H relative to C activity genotype. A study of cerebellar samples reported associations between polymorphisms in Chrm1 and modifications in aspects of executive function in humans [113]. This gene is related to dopamine transmission [114, 115] and further supports the notion that mice from the studied high activity genotype share disruption of dopamine pathways with drug and other reward-dependent behaviors. Other genes over-expressed in mice from the H relative to C activity genotype include the transcription factor Forkhead Box G1 (Foxg1). Studies of cerebellar granule neurons report that this gene promotes neuronal survival [116]. Differential expression of endothelial Lipase (Lipg) between the H and C genotypes was observed. Lipg is associated with neurite pathology and is detected in the cerebellum [117]. The association with addictive behaviors of these genes differentially expressed between H and C activity genotypes highlights the genetic pathways shared between high voluntary activity and neurological, cognitive, and addiction processes.

Results from the functional enrichment analysis of the genes differentially (FDR-adjusted P-value < 0.05) expressed between activity genotypes are consistent with previous findings about processes in the cerebellum associated with locomotor control and offer additional evidence on the link between high activity genotype and reward-dependent behaviors [1]. Terms enriched included voltage-gated cation channel activity (Enrichment Score = 5.36), GTPase regulatory activity (Enrichment Score = 2.04), neuroactive ligand-receptor interaction (Enrichment Score = 1.73), metal ion binding (Enrichment Score = 1.59), regulation of cAMP metabolic process (Enrichment Score = 1.48), neurotransmitter receptor activity (Enrichment Score = 1.25), and neuron projection morphogenesis (Enrichment Score = 1.18). The most significantly enriched cluster is highlighted in Table 4. The majority of the terms were related to ion binding and activity. This result is consistent with a study of working memory-related activity in the cerebellum that uncovered enrichment of voltage-gated cation channel activity encompassing genes related to neuronal excitability [118]. Working memory is the active maintenance of task-relevant information during a cognitive task and is expected to be important in activities such as wheel running. Our result suggests that selective breeding for high activity has changed the expression of a number of transcripts, many of which are not environment-dependent (e.g. Drd1 and Chrm1). These genes have been annotated to processes supporting motivation or addiction-like behavior towards activity, including signaling and locomotor control, thus enabling the mice to execute high intensity exercise. Some of these processes are shared with genes that exhibit environment-dependent differential expression between genotypes. Therefore, conclusions from systems biology studies of high voluntary running lines should consider the context or environment of the study.

**Table 4:** Enriched (enrichment score = 5.36) cluster of Gene Ontology (GO) biological process (BP), molecular function (MF) Functional Annotation Tool (FAT) categories among the genes significantly differentially expressed (FDR-adjusted P-value < 0.05) between mice from Control (C) and High (H) activity genotypes.

Category	Term	Count	P-value	FDR P-value
Score	= 5.36			
MF	GO:0022843~voltage-gated cation channel activity	13	6.43E-09	8.50E-06
MF	GO:0022832~voltage-gated channel activity	14	3.26E-08	4.31E-05
MF	GO:0005244~voltage-gated ion channel activity	14	3.26E-08	4.31E-05
MF	GO:0005261~cation channel activity	15	2.21E-07	2.92E-04
BP	GO:0030001~metal ion transport	19	3.35E-07	5.21E-04
BP	GO:0006812~cation transport	20	6.92E-07	1.08E-03
MF	GO:0005249~voltage-gated potassium channel activity	10	7.05E-07	9.32E-04
MF	GO:0046873~metal ion transmembrane transporter activity	15	1.45E-06	1.92E-03
MF	GO:0022836~gated channel activity	14	5.61E-06	7.42E-03
MF	GO:0005267~potassium channel activity	10	6.46E-06	8.54E-03
MF	GO:0005216~ion channel activity	15	1.24E-05	1.63E-02
MF	GO:0022838~substrate specific channel activity	15	1.75E-05	2.31E-02
MF	GO:0015267~channel activity	15	2.04E-05	2.70E-02
MF	GO:0022803~passive transmembrane transporter activity	15	2.04E-05	2.70E-02
BP	GO:0006811~ion transport	21	2.13E-05	3.31E-02
MF	GO:0030955~potassium ion binding	9	2.64E-05	3.49E-02
BP	GO:0006813~potassium ion transport	10	2.89E-05	4.49E-02
MF	GO:0031420~alkali metal ion binding	11	4.72E-05	6.23E-02
BP	GO:0015672~monovalent inorganic cation transport	12	2.04E-04	3.17E-01

### Effect of activity environment on gene expression in the cerebellum

Differential expression patterns between activity environments that are genotype-independent were uncovered in addition to genotype-dependent patterns. Table 5 summarizes genes differentially expressed (log2(fold change) > |4|, FDR-adjusted P-value < 0.007) between the B and F environments. A more complete list of genes differentially expressed at FDR-adjusted P-value < 0.05 is provided within **Table D** in S1 File. The majority of the 148 differentially expressed genes in the cerebellum were under-expressed in the B relative to the F environment mice. Moreover, many of the genes under-expressed in the B relative to the F environment groups (e.g. Foxg1, Prss56, Dlx1) were also under-expressed in the C relative to the H genotype mice.

**Table 5:** Genes significantly differentially expressed (FDR-adjusted P-value < 0.007) and log2(fold change) > |4|) between mice in Blocked (B) and Free (F) activity environments.

Gene Symbol	Gene Name	UCSC ID^a^	Log2 (B/F)	FDR P-value
Foxg1	forkhead box G1	uc007nmf.2/uc011ylr.1	-6.58	0.007
Dlx1	distal-less homeobox 1	uc008kau.1/uc008kav.1	-6.31	0.007
Kcnv1	potassium channel, subfamily V, member 1	uc007vql.2/uc007vqm.2	-5.66	0.007
Drd1	dopamine receptor D1	uc011yzm.2	-5.47	0.007
Zfp831	zinc finger protein 831	uc008ofh.1	-5.30	0.007
Sp9	trans-acting transcription factor 9	uc008kcj.2	-5.23	0.007
Gpr6	G protein-coupled receptor 6	uc007exj.1	-5.23	0.007
Kcnj4	potassium inwardly-rectifying channel, subfamily J, member 4	uc007wtn.2	-5.10	0.007
Chrm1	cholinergic receptor, muscarinic 1, CNS	uc008gmf.2/uc008gmg.2	-5.08	0.007
Ankrd63	ankyrin repeat domain 63	uc008lsh.1	-5.08	0.007
Gda	guanine deaminase	uc008gyx.1	-5.03	0.007
Krt9	keratin 9	uc007lkn.1	-4.96	0.007
Lrrc10b	leucine rich repeat containing 10B	uc012bio.2	-4.82	0.007
Cpne5	copine V	uc008bsj.2/uc008bsk.2	-4.78	0.007
Kcnf1	potassium voltage-gated channel, subfamily F, member 1	uc007ncn.2	-4.78	0.007
Rxrg	retinoid X receptor gamma	uc007dla.2/uc007dlb.2	-4.66	0.007
Kcng1	potassium voltage-gated channel, subfamily G, member 1	uc008oat.1	-4.56	0.007
Tbr1	T-box brain gene 1	uc008jvd.1	-4.18	0.007
Cntnap3	contactin associated protein-like 3	uc007qyy.1/uc007qyz.1	-4.17	0.007
Adora2a	adenosine A2a receptor	uc007fqh.1	-4.15	0.007
Crhbp	corticotropin releasing hormone binding protein	uc007rmk.2	-4.02	0.007

^a^ University of California Santa Cruz genome browser identifier.

The correlation between the log2(fold changes) of the 123 genes both differentially expressed between activity genotypes (**Table C** in S1 File) and between activity environments (**Table D** in S1 File) was 0.98. Furthermore, among the 207 genes differentially expressed in either comparison (genotype or environment) the correlation in log2(fold change) ranged between 0.81 and 0.84. The similar gene expression profiles between the C—H and B—F contrasts indicate that the genotypes for activity reward compared in this study are a robust model to study the impact of reward availability in the environment on the cerebellum transcriptome, provided the genes do not exhibit significant interaction effects.

Terms enriched among differentially expressed genes between activity availability environments were similar to the terms identified for the genotype contrast and included voltage-gated cation channel activity (Enrichment Score = 5.43), neuroactive ligand-receptor interaction (Enrichment Score = 2.77), neuron projection morphogenesis (Enrichment Score = 2.11), GTPase regulatory activity (Enrichment Score = 1.79), regulation of cAMP metabolic process (Enrichment Score = 1.44), positive regulation of transcription (Enrichment Score = 1.11), and metal ion binding (Enrichment Score = 0.99). A notable finding is that neurotransmitter receptor activity did not reach enrichment level in the environment contrast unlike in the genotype contrast. The most enriched cluster is highlighted in Table 6.

**Table 6:** Enriched (enrichment score = 5.43) clusters of Gene Ontology (GO) biological process (BP) and molecular function (MF) Functional Annotation Tool (FAT) categories among the genes significantly differentially expressed (P-value < 0.001) between mice in Blocked (B) and Free (F) activity environments.

Category	GO Term	Count	P-value	FDR P-value
Score	= 5.43			
MF	GO:0022843~voltage-gated cation channel activity	12	4.97E-09	6.41E-06
MF	GO:0005249~voltage-gated potassium channel activity	11	5.33E-09	6.88E-06
MF	GO:0005267~potassium channel activity	12	5.40E-09	6.97E-06
BP	GO:0006813~potassium ion transport	12	3.03E-08	4.57E-05
MF	GO:0022832~voltage-gated channel activity	12	1.62E-07	2.09E-04
MF	GO:0005244~voltage-gated ion channel activity	12	1.62E-07	2.09E-04
MF	GO:0005261~cation channel activity	13	5.75E-07	7.43E-04
MF	GO:0022836~gated channel activity	13	2.14E-06	0.0027684
MF	GO:0046873~metal ion transmembrane transporter activity	13	2.97E-06	0.0038393
MF	GO:0030955~potassium ion binding	9	4.14E-06	0.0053489
BP	GO:0015672~monovalent inorganic cation transport	12	1.66E-05	0.0250304
MF	GO:0005216~ion channel activity	13	1.94E-05	0.0250603
BP	GO:0030001~metal ion transport	14	2.57E-05	0.0387331
MF	GO:0022838~substrate specific channel activity	13	2.64E-05	0.0340420
BP	GO:0006812~cation transport	15	2.94E-05	0.0442644
MF	GO:0022803~passive transmembrane transporter activity	13	3.02E-05	0.0389763
MF	GO:0015267~channel activity	13	3.02E-05	0.0389763
MF	GO:0031420~alkali metal ion binding	9	2.24E-04	0.2891100

### Motivation towards high activity and addiction processes

Reward-motivated responses, such as those associated with addictive behaviors, are regulated by the dopaminergic mesocorticolimbic system in the brain [119–121]. Substances of abuse, including opiates, cocaine, amphetamine, alcohol, and nicotine, are addictive pleasurable stimuli that impact the neurotransmission of dopamine in a manner similar to other more natural stimuli including specific foods or liquids, gambling, shopping, and exercise or running [15, 17, 122–129]. While addiction to exercise is not considered an official addictive disorder [130], 3% to 5% of the US population experience this condition [131].

Our investigation of cerebellum transcriptomic differences between mice from high wheel running and control genotypes in environments enabling and blocking access to wheel running uncovered enrichment of dopamine and related signaling pathways, and neurotransmitter and neuropeptide-dependent processes. Dysregulation of genes in these processes have also been linked to reward-dependent behaviors. Our findings support the premise that the line selected for high running and motivated to run studied here can be an effective model to study the transcriptome associated with addictive disorders.

## Conclusions

Comparison of the cerebellum transcriptome between H (High Runner) and C (Control) mouse lines in environments with F (free) or B (blocked) access to a running wheel on day 7 provided novel insights into environment dependent and independent differential gene expression and networks. Overall, 457 genes exhibited significant (FDR-adjusted P-value < 0.05) activity genotype-by-environment interaction effect. The number of genes differentially expressed in the orthogonal contrast between genotype-environment groups CB-HB, CB-CF, and CF-HF were 233, 225, and 248, respectively. These genes included Pak6, Adora2a, Drd2, and Arhgap8, neuropeptide prohormones: Adcyap1, Cck, Sst, Vgf, Npy, Nts, Penk, and Tac2 and related receptors: Gcgr, Vipr1, Vipr2, and Crhr2. These findings support the need to characterize transcriptome dysregulation between high voluntary running and control lines in an environment or context-dependent manner. This recommendation may be relevant to other reward-dependent behaviors.

The networks of the CB-HB and CF-HF contrasts exhibited extreme and opposite gene expression profiles and the differentially expressed genes were more connected in the CF-HF network. This suggest that expression changes in highly connected genes in the cerebellum of H genotype mice may be associated with the capacity of these mice to act upon their motivation to run and to execute high wheel running in the F environment.

The majority of the 183 differentially expressed genes between genotypes (including Drd1) were under-expressed in the cerebellum of C relative to H genotype mice. Also, the majority of the 148 differentially expressed genes were under-expressed in the cerebellum of mice in the B relative to F activity environment. Of these, 123 genes were differentially expressed in both comparisons.

Drug induced activity-dependent synaptic modifications in the cerebellum have been postulated to be key in the transition from a pattern of recreational drug taking to the compulsive behavioral phenotype [1]. This characterization is complemented by our study of the cerebellum that identified changes in the expression of genes associated with the motivation and reward-dependent behaviors in a mouse line selected for high running.

Our findings demonstrate that the cerebellum of a mouse line selected for high voluntary running share transcriptome patterns with reward-dependent behaviors, such as addiction to psychoactive substances. Significant changes in the expression of genes affiliated to dopamine-related and neuropeptide-dependent processes and their association with motivation and reward dependent behaviors and locomotor control were explored. The high voluntary activity selection line of mice evaluated in this study is a helpful model for understanding molecular mechanisms behind motivation, reward-dependent, and addictive behaviors.

## Supporting Information

S1 File Supplementary results. **Table A.** Genes exhibiting significant (FDR-adjusted P-value < 0.05) activity genotype-by-environment interaction and Log2(Fold Change) by pairwise contrast encompassing genotype (C and H) and environment (B and F) groups. **Table B.** Enriched clusters of Gene Ontology (GO) biological process (BP), molecular function (MF) Functional Annotation Tool (FAT) categories, and KEGG pathways among the genes that exhibited significant genotype-by-environment interaction (FDR-adjusted P-value < 0.05) and differentially expressed (FDR-adjusted P-value < 0.05) between mice from genotype-by-environment groups in the 3 orthogonal contrasts (CB-HB, CF-HF, and HB-HF). **Table C.** Genes differentially expressed (FDR-adjusted P-value < 0.05) between mice from the Control and High activity genotypes. **Table D.** Genes differentially expressed (FDR-adjusted P-value < 0.05) between mice in Blocked and Free activity environments.
(DOCX)
