# Supplementary material for: Cerebellum Transcriptome of Mice Bred for High Voluntary Activity Offers Insights into Locomotor Control and Reward-Dependent Behaviors
Source: PLoS One. 2016 Nov 28;11(11):e0167095. doi: 10.1371/journal.pone.0167095 (PMC5125674; doi:10.1371/journal.pone.0167095)
Supplement: S1 File — Table A. Genes exhibiting significant (FDR-adjusted P-value < 0.05) activity genotype-by-environment interaction and Log2(Fold Change) by pairwise contrast encompassing genotype (C and H) and environment (B and F) groups. Table B. Enriched clusters of Gene Ontology (GO) biological process (BP), molecular function (MF) Functional Annotation Tool (FAT) categories, and KEGG pathways among the genes that exhibited significant genotype-by-environment interaction (FDR-adjusted P-value < 0.05) and differentially expressed (FDR-adjusted P-value < 0.05) between mice from genotype-by-environment groups in the 3 orthogonal contrasts (CB-HB, CF-HF, and HB-HF). Table C. Genes differentially expressed (FDR-adjusted P-value < 0.05) between mice from the Control and High activity genotypes. Table D. Genes differentially expressed (FDR-adjusted P-value < 0.05) between mice in Blocked and Free activity environments. (DOCX) [file pone.0167095.s001.docx]

**Table A.** Genes exhibiting significant (FDR-adjusted P-value < 0.05) activity genotype-by-environment interaction and log2(Fold Change) by pairwise contrast encompassing genotype (C and H) and environment (B and F) groups.

| **Gene Symbol** |  |  | **Log2(Fold Change)^1^** |  |  |  | **FDR-adjusted P-value** |
| --- | --- | --- | --- | --- | --- | --- | --- |
|  | **CB-HB** | **CB-CF** | **CB-HF** | **HB-CF** | **HB-HF** | **CF-HF** |  |
| Ddn | -5.1239 | -7.6548 | -6.92317 | -2.5309 | -1.79927 | 0.731627 | 2.57307E-14 |
| Gpr88 | -4.48439 | -6.42635 | -5.93863 | -1.94196 | -1.45425 | 0.487719 | 2.57307E-14 |
| Icam5 | -4.57647 | -6.21878 | -5.7006 | -1.64231 | -1.12413 | 0.518182 | 2.57307E-14 |
| Gda | -4.60835 | -6.54463 | -5.29571 | -1.93628 | -0.687357 | 1.24892 | 2.57307E-14 |
| Kcnj4 | -4.596 | -6.17144 | -5.47734 | -1.57544 | -0.881339 | 0.694103 | 2.57307E-14 |
| Egr3 | -4.49046 | -5.92918 | -5.50059 | -1.43872 | -1.01013 | 0.428586 | 2.57307E-14 |
| Lrrc10b | -4.43931 | -5.64017 | -5.25873 | -1.20086 | -0.819418 | 0.38144 | 2.57307E-14 |
| Cpne5 | -4.54153 | -5.76022 | -4.817 | -1.21869 | -0.275474 | 0.943217 | 2.57307E-14 |
| Zfp33b | 2.36733 | -3.81808 | 2.4553 | -6.18542 | 0.0879719 | 6.27339 | 2.57307E-14 |
| Nrgn | -4.50147 | -5.50741 | -4.02525 | -1.00594 | 0.47622 | 1.48216 | 2.57307E-14 |
| Rxrg | -3.84299 | -5.2896 | -4.36536 | -1.44661 | -0.522372 | 0.924239 | 2.57307E-14 |
| Tbr1 | -3.99168 | -4.93814 | -4.6618 | -0.946458 | -0.670122 | 0.276336 | 2.57307E-14 |
| Adora2a | -4.02261 | -4.8696 | -4.72083 | -0.846991 | -0.698221 | 0.14877 | 2.57307E-14 |
| Drd2 | -4.18193 | -4.83802 | -4.09709 | -0.65609 | 0.0848317 | 0.740922 | 2.57307E-14 |
| Scd4 | 3.28115 | -1.66477 | 3.17214 | -4.94592 | -0.109003 | 4.83692 | 2.57307E-14 |
| Lamp5 | -2.9593 | -4.53335 | -3.87565 | -1.57405 | -0.916353 | 0.657694 | 2.57307E-14 |
| Sst | -4.16424 | -4.60927 | -3.54259 | -0.445032 | 0.621649 | 1.06668 | 2.57307E-14 |
| Ctxn1 | -3.84723 | -4.59672 | -4.05427 | -0.749491 | -0.207042 | 0.54245 | 2.57307E-14 |
| Cpne4 | -3.45958 | -4.77135 | -3.56315 | -1.31177 | -0.103572 | 1.2082 | 2.57307E-14 |
| D430019H16Rik | -3.63946 | -4.27087 | -3.72426 | -0.631411 | -0.0848064 | 0.546604 | 2.57307E-14 |
| Lpl | -3.31099 | -4.339 | -3.40651 | -1.02801 | -0.095513 | 0.932495 | 2.57307E-14 |
| Pak6 | -3.48171 | -4.07743 | -3.12135 | -0.595715 | 0.360363 | 0.956078 | 2.57307E-14 |
| Mpped1 | -3.04605 | -3.62686 | -3.10446 | -0.580813 | -0.0584062 | 0.522406 | 2.57307E-14 |
| Padi4 | 2.74917 | -0.758261 | 1.63504 | -3.50743 | -1.11413 | 2.39331 | 2.57307E-14 |
| Pcdh8 | -2.27045 | -2.57307 | -2.05065 | -0.302618 | 0.219808 | 0.522427 | 2.57307E-14 |
| Nsg2 | -1.92034 | -1.0907 | -1.8603 | 0.829641 | 0.0600423 | -0.769599 | 2.57307E-14 |
| Dnah1 | -1.80707 | -2.00622 | -1.6527 | -0.199149 | 0.15437 | 0.35352 | 2.57307E-14 |
| Gm1976 | 0.0160057 | -2.56793 | -0.128706 | -2.58394 | -0.144712 | 2.43922 | 2.57307E-14 |
| Rpl35a | 1.03626 | -1.05853 | 0.854784 | -2.0948 | -0.181479 | 1.91332 | 2.57307E-14 |
| Slc37a1 | 0.392008 | -1.56303 | 0.741699 | -1.95504 | 0.349691 | 2.30473 | 2.57307E-14 |
| Spp1 | 1.36966 | -0.0880643 | 1.17762 | -1.45773 | -0.19204 | 1.26569 | 2.57307E-14 |
| 4933409K07Rik | -1.03756 | -0.367354 | -1.47545 | 0.670202 | -0.437892 | -1.10809 | 2.57307E-14 |
| Mmd | -1.03162 | -1.3325 | -1.06407 | -0.30088 | -0.0324504 | 0.26843 | 2.57307E-14 |
| Cox7b | 0.919044 | -0.212447 | 0.97375 | -1.13149 | 0.0547066 | 1.1862 | 2.57307E-14 |
| Zfp605 | -0.958906 | 0.134878 | -0.980946 | 1.09378 | -0.0220399 | -1.11582 | 2.57307E-14 |
| Slco2a1 | -0.708232 | 0.52378 | -0.621142 | 1.23201 | 0.0870896 | -1.14492 | 2.57307E-14 |
| Tpm3 | 0.557521 | -0.673741 | 0.596702 | -1.23126 | 0.0391806 | 1.27044 | 2.57307E-14 |
| Adi1 | 0.894847 | -0.129189 | 0.93957 | -1.02404 | 0.0447231 | 1.06876 | 2.57307E-14 |
| Cntn4 | 0.747304 | -0.157213 | 0.583302 | -0.904517 | -0.164002 | 0.740515 | 2.57307E-14 |
| Gsn | 0.281971 | -0.125945 | 0.341209 | -0.407916 | 0.0592376 | 0.467154 | 2.57307E-14 |
| Mtcl1 | 0.241729 | 0.322627 | 0.254441 | 0.0808978 | 0.0127112 | -0.0681866 | 2.57307E-14 |
| Ppp2r5c | -0.269808 | -0.11696 | -0.211833 | 0.152848 | 0.0579742 | -0.0948736 | 2.57307E-14 |
| Gfra2 | -0.818719 | -1.19728 | -0.722761 | -0.37856 | 0.0959582 | 0.474518 | 7.36835E-14 |
| Spats2l | -0.413439 | -0.675313 | -0.40825 | -0.261874 | 0.00518901 | 0.267063 | 7.36835E-14 |
| Srgap2 | -0.0322648 | 0.234135 | -0.0485468 | 0.2664 | -0.016282 | -0.282682 | 1.68108E-13 |
| Gcnt1 | 1.08573 | 0.270775 | 0.846821 | -0.814953 | -0.238908 | 0.576045 | 3.52399E-13 |
| Gpr83 | -2.92761 | -3.71396 | -3.13102 | -0.786355 | -0.203411 | 0.582944 | 6.43816E-13 |
| Pcsk9 | 0.863776 | -2.91167 | 0.361879 | -3.77545 | -0.501897 | 3.27355 | 7.88004E-13 |
| Mybpc3 | -0.730238 | 0.0950949 | -1.05062 | 0.825333 | -0.320386 | -1.14572 | 1.10275E-12 |
| Hs3st4 | -3.37427 | -4.16933 | -3.20967 | -0.795065 | 0.164593 | 0.959658 | 2.87464E-12 |
| Asic4 | -3.56982 | -4.32922 | -3.05823 | -0.759404 | 0.511588 | 1.27099 | 2.98779E-12 |
| Fam19a2 | -1.57192 | -1.691 | -1.62744 | -0.119087 | -0.0555265 | 0.0635603 | 3.22129E-12 |
| Sostdc1 | 2.32082 | 0.345433 | 2.00047 | -1.97539 | -0.320352 | 1.65504 | 3.87418E-12 |
| Nat8f2 | 1.28336 | -1.74923 | 0.862648 | -3.03259 | -0.420715 | 2.61188 | 3.94252E-12 |
| Stk32c | -2.89526 | -3.3837 | -2.94497 | -0.488437 | -0.0497104 | 0.438727 | 4.72802E-12 |
| Pde1b | -2.49235 | -2.85695 | -2.52922 | -0.364593 | -0.0368683 | 0.327725 | 4.72802E-12 |
| Pisd-ps3 | -1.21636 | -0.231725 | -1.0878 | 0.984638 | 0.128558 | -0.856079 | 5.61201E-12 |
| Kif19a | 1.53581 | -0.452659 | 1.33116 | -1.98847 | -0.204653 | 1.78382 | 5.62705E-12 |
| Atrnl1 | 0.0592132 | -0.302828 | 0.0141022 | -0.362041 | -0.0451109 | 0.31693 | 6.83217E-12 |
| Gm266 | 0.969712 | -1.63121 | 1.12245 | -2.60093 | 0.152735 | 2.75366 | 1.02486E-11 |
| Scn4b | -0.970815 | -1.71178 | -1.05382 | -0.740965 | -0.0830095 | 0.657956 | 1.6671E-11 |
| Pgap2 | 0.314493 | 0.278323 | 0.402003 | -0.0361699 | 0.0875093 | 0.123679 | 1.74479E-11 |
| Syndig1l | -2.08479 | -1.59212 | -1.96082 | 0.492671 | 0.123977 | -0.368694 | 2.05846E-11 |
| Enc1 | -3.18691 | -3.50262 | -3.18764 | -0.315714 | -0.000733497 | 0.31498 | 2.07189E-11 |
| Neto1 | -3.11794 | -3.66169 | -3.30468 | -0.543753 | -0.186744 | 0.357009 | 2.0982E-11 |
| Arhgap8 | 2.06692 | -3.50808 | 2.25669 | -5.575 | 0.189776 | 5.76478 | 2.31366E-11 |
| Wdr17 | -1.33581 | -1.26227 | -1.40603 | 0.0735414 | -0.0702167 | -0.143758 | 3.55015E-11 |
| Nell1 | 0.470607 | -0.186662 | 0.387923 | -0.657268 | -0.0826838 | 0.574585 | 5.64343E-11 |
| A230057D06Rik | 0.432103 | 0.128195 | 0.132177 | -0.303908 | -0.299926 | 0.00398154 | 6.51077E-11 |
| Mal2 | -2.60886 | -3.28445 | -2.78545 | -0.675589 | -0.176592 | 0.498996 | 6.5598E-11 |
| Khdrbs3 | -1.41039 | -1.39757 | -1.26189 | 0.0128235 | 0.148506 | 0.135682 | 6.73225E-11 |
| Camk2a | -2.45949 | -2.95646 | -2.60341 | -0.496977 | -0.14392 | 0.353057 | 9.35698E-11 |
| Hpcal4 | -2.69367 | -3.15287 | -2.84522 | -0.459197 | -0.151551 | 0.307646 | 1.3442E-10 |
| Necab2 | -2.29869 | -3.2822 | -2.08322 | -0.98351 | 0.21547 | 1.19898 | 1.4271E-10 |
| Ryr3 | -0.29519 | -0.903104 | -0.403508 | -0.607914 | -0.108317 | 0.499597 | 1.68458E-10 |
| Akap5 | -2.55117 | -3.09202 | -2.78657 | -0.540853 | -0.235409 | 0.305444 | 1.80987E-10 |
| Abcb8 | -0.786506 | 0.088069 | -0.91637 | 0.874575 | -0.129864 | -1.00444 | 1.89626E-10 |
| Rprml | -4.03024 | -3.78015 | -4.3396 | 0.250085 | -0.309358 | -0.559444 | 2.7408E-10 |
| AK036371 | 1.43424 | 0.150169 | 1.417 | -1.28407 | -0.0172397 | 1.26683 | 3.59241E-10 |
| Kcnh3 | -2.60524 | -2.56092 | -2.73757 | 0.044316 | -0.132328 | -0.176644 | 3.75918E-10 |
| Cyp11a1 | 1.22272 | -1.37787 | 1.26116 | -2.60059 | 0.0384445 | 2.63903 | 4.24218E-10 |
| Gm10419 | -0.663488 | -1.21864 | -0.880177 | -0.555148 | -0.21669 | 0.338458 | 5.61235E-10 |
| Nptx2 | -2.3725 | -3.71948 | -2.42485 | -1.34698 | -0.0523535 | 1.29463 | 6.23507E-10 |
| Vgf | -1.32199 | -2.14466 | -1.44074 | -0.822678 | -0.118754 | 0.703924 | 6.49084E-10 |
| Rwdd3 | -0.846542 | -0.288251 | -1.02295 | 0.558291 | -0.176411 | -0.734702 | 8.05687E-10 |
| Dnah7b | -0.934535 | -0.0262482 | -0.963088 | 0.908287 | -0.0285529 | -0.93684 | 8.88366E-10 |
| Aplp2 | 0.14101 | 0.147453 | 0.127828 | 0.00644283 | -0.0131824 | -0.0196253 | 9.15519E-10 |
| Adam1a | -0.162796 | 0.232089 | -0.180903 | 0.394885 | -0.0181068 | -0.412992 | 1.21413E-09 |
| Gng7 | -3.021 | -3.29534 | -3.3112 | -0.274341 | -0.290202 | -0.0158609 | 1.31934E-09 |
| Rps19 | 0.259041 | -1.37869 | -0.0656595 | -1.63773 | -0.324701 | 1.31303 | 2.04314E-09 |
| Mdfic | 0.491936 | 0.0102914 | 0.195502 | -0.481645 | -0.296434 | 0.185211 | 2.26594E-09 |
| Mchr1 | -3.43815 | -3.96044 | -3.74797 | -0.522284 | -0.309816 | 0.212468 | 2.51473E-09 |
| Fam124a | -1.52712 | -0.143807 | -1.49275 | 1.38331 | 0.0343681 | -1.34894 | 2.86732E-09 |
| Rab40b | -3.24232 | -3.69485 | -3.174 | -0.452529 | 0.0683216 | 0.520851 | 4.06536E-09 |
| Gcgr | 1.81582 | -0.170279 | 1.34061 | -1.9861 | -0.475212 | 1.51089 | 5.89163E-09 |
| AK148766 | 0.864152 | -3.5235 | 0.747599 | -4.38765 | -0.116552 | 4.2711 | 6.26678E-09 |
| Basp1 | -2.84579 | -3.01903 | -2.6405 | -0.173237 | 0.205287 | 0.378524 | 6.26678E-09 |
| GH | 0.0933079 | 3.59206 | 0.912204 | 3.49875 | 0.818896 | -2.67986 | 9.2265E-09 |
| Gjc3 | -0.623022 | 0.114987 | -0.743677 | 0.738009 | -0.120655 | -0.858664 | 1.38518E-08 |
| Mob3b | 0.97277 | 0.0462873 | 0.924868 | -0.926483 | -0.0479022 | 0.878581 | 2.3242E-08 |
| Chil5 | 0.870484 | -0.0543656 | 1.09069 | -0.924849 | 0.220206 | 1.14506 | 2.34114E-08 |
| Stom | 1.11657 | 0.0703515 | 1.17307 | -1.04622 | 0.0564941 | 1.10271 | 3.27368E-08 |
| Actn2 | -3.51744 | -3.3538 | -4.21886 | 0.163646 | -0.701415 | -0.86506 | 3.5721E-08 |
| Nts | -3.36256 | -4.72986 | -3.36298 | -1.36731 | -0.000425478 | 1.36688 | 5.2147E-08 |
| Adcyap1 | -2.29727 | -0.545307 | -1.88834 | 1.75196 | 0.408927 | -1.34304 | 5.28525E-08 |
| Slit1 | -3.3458 | -3.52019 | -2.96096 | -0.174384 | 0.384849 | 0.559233 | 5.38783E-08 |
| Gm11711 | 2.55356 | -0.0484632 | 2.66339 | -2.60202 | 0.109829 | 2.71185 | 7.18327E-08 |
| Fscn1 | -1.48735 | -0.854181 | -1.57844 | 0.633165 | -0.0910925 | -0.724257 | 8.84656E-08 |
| 6030419C18Rik | 0.136956 | -0.39986 | 0.176153 | -0.536816 | 0.0391974 | 0.576013 | 1.17855E-07 |
| Gng4 | -3.12964 | -3.19848 | -3.13436 | -0.0688357 | -0.00472437 | 0.0641113 | 1.35588E-07 |
| March1 | -1.55526 | -1.88322 | -1.64848 | -0.327967 | -0.0932207 | 0.234747 | 1.40566E-07 |
| Aldh1a1 | 0.839945 | 0.227631 | 0.978124 | -0.612313 | 0.13818 | 0.750493 | 1.52246E-07 |
| Krt25 | -1.81047 | 0.174426 | -1.78743 | 1.9849 | 0.0230466 | -1.96185 | 1.66412E-07 |
| Rgs4 | -2.50085 | -2.54107 | -2.59207 | -0.0402172 | -0.0912174 | -0.0510002 | 1.74223E-07 |
| Rin1 | -2.84324 | -2.79399 | -3.26406 | 0.0492477 | -0.420822 | -0.470069 | 1.77086E-07 |
| Lamc2 | 1.04948 | -0.792898 | 0.911649 | -1.84238 | -0.137835 | 1.70455 | 1.98955E-07 |
| Dbpht2 | -2.87797 | -3.25805 | -3.12506 | -0.380076 | -0.247086 | 0.132991 | 2.08402E-07 |
| Ccnd2 | -1.8165 | -2.1434 | -1.78425 | -0.326899 | 0.0322546 | 0.359153 | 2.14871E-07 |
| Trub1 | -0.606145 | -0.225609 | -0.76572 | 0.380536 | -0.159575 | -0.540111 | 2.19234E-07 |
| Rps6kl1 | -0.400929 | -0.214725 | -0.337318 | 0.186205 | 0.0636111 | -0.122593 | 2.32421E-07 |
| Gm14403 | 0.245507 | -0.82502 | 0.364498 | -1.07053 | 0.118991 | 1.18952 | 2.3867E-07 |
| Mcm6 | -1.89114 | 0.0575242 | -1.65909 | 1.94866 | 0.232044 | -1.71662 | 2.61952E-07 |
| Cacna1h | -2.5653 | -2.93281 | -2.66178 | -0.36751 | -0.0964842 | 0.271026 | 2.6494E-07 |
| Col19a1 | 0.19112 | -0.913692 | -0.115188 | -1.10481 | -0.306308 | 0.798505 | 2.74283E-07 |
| Arl5a | 0.627926 | 0.010811 | 0.593557 | -0.617115 | -0.0343692 | 0.582746 | 2.74283E-07 |
| Plcd4 | 0.561339 | -0.0402928 | 0.482159 | -0.601632 | -0.0791805 | 0.522452 | 2.74283E-07 |
| Hebp2 | 1.46014 | -0.214492 | 1.57081 | -1.67463 | 0.110668 | 1.7853 | 2.9866E-07 |
| Plppr1 | -2.94418 | -3.26185 | -3.17815 | -0.317669 | -0.233971 | 0.0836979 | 3.67093E-07 |
| AK133650 | -2.19713 | 0.170843 | -2.43552 | 2.36797 | -0.238399 | -2.60637 | 5.80619E-07 |
| AK134928 | 0.572809 | -1.49332 | 0.315691 | -2.06613 | -0.257118 | 1.80901 | 5.99669E-07 |
| Fam163b | -2.20994 | -2.62346 | -1.99951 | -0.413518 | 0.210426 | 0.623944 | 6.94893E-07 |
| Tmem132b | -1.97035 | -2.35463 | -2.11522 | -0.384275 | -0.144863 | 0.239412 | 7.42683E-07 |
| Cdh9 | -3.24721 | -3.5984 | -2.61143 | -0.351183 | 0.635786 | 0.986969 | 7.44565E-07 |
| Sycp1 | 0.897514 | 0.0438019 | 0.912743 | -0.853712 | 0.0152291 | 0.868941 | 7.60129E-07 |
| Noc2l | 0.304161 | -0.228696 | 0.354742 | -0.532857 | 0.0505806 | 0.583437 | 7.76322E-07 |
| Cyp2j12 | 1.10256 | -0.418553 | 0.781681 | -1.52111 | -0.32088 | 1.20023 | 8.4379E-07 |
| Col13a1 | 0.968822 | -0.0973093 | 0.698149 | -1.06613 | -0.270673 | 0.795458 | 8.48856E-07 |
| Ephb6 | -1.43103 | -2.0397 | -1.47615 | -0.608668 | -0.0451131 | 0.563555 | 1.07861E-06 |
| Creg2 | -2.08055 | -2.54364 | -2.11547 | -0.463093 | -0.0349269 | 0.428166 | 1.13853E-06 |
| 6330403A02Rik | -2.32261 | -2.47813 | -2.46622 | -0.155518 | -0.143608 | 0.0119096 | 1.2286E-06 |
| Npy | -1.2674 | -2.2958 | -1.04529 | -1.02839 | 0.222113 | 1.25051 | 1.29839E-06 |
| 2610305D13Rik | 3.88408 | 1.79244 | 2.73734 | -2.09165 | -1.14674 | 0.944904 | 1.44426E-06 |
| Adcy5 | -1.89602 | -2.17723 | -2.00222 | -0.281209 | -0.106203 | 0.175005 | 1.64387E-06 |
| Cxcr4 | 0.942661 | -0.161189 | 0.944332 | -1.10385 | 0.00167133 | 1.10552 | 1.64387E-06 |
| Rps3a1 | 0.476418 | -0.463548 | 0.559907 | -0.939966 | 0.0834894 | 1.02346 | 1.66362E-06 |
| Gucy1a2 | 0.324706 | -0.347554 | 0.316043 | -0.67226 | -0.00866286 | 0.663597 | 1.7457E-06 |
| Slc35d3 | -3.23705 | -3.41352 | -3.6886 | -0.176465 | -0.451547 | -0.275081 | 2.02487E-06 |
| Inadl | -0.204585 | 0.366736 | -0.105116 | 0.571321 | 0.0994693 | -0.471852 | 2.43459E-06 |
| Foxj1 | -0.491259 | -0.631787 | -0.513539 | -0.140528 | -0.02228 | 0.118248 | 2.52824E-06 |
| Pou3f1 | -2.30866 | -2.83142 | -2.56184 | -0.52276 | -0.253172 | 0.269588 | 2.55442E-06 |
| Tmem158 | -1.85645 | -2.32905 | -1.90374 | -0.472602 | -0.0472847 | 0.425317 | 2.82913E-06 |
| Prkcd | -0.218913 | 0.0896678 | -0.200955 | 0.308581 | 0.0179583 | -0.290623 | 3.28098E-06 |
| Kcna4 | -1.98649 | -2.76253 | -2.34525 | -0.77604 | -0.358761 | 0.417279 | 4.20558E-06 |
| Syt5 | -1.92426 | -2.36959 | -1.7692 | -0.445333 | 0.15506 | 0.600393 | 4.27016E-06 |
| A830018L16Rik | -1.09165 | -1.1783 | -1.10521 | -0.0866559 | -0.0135609 | 0.073095 | 4.35059E-06 |
| Exd2 | 0.0574492 | -0.0256191 | 0.0456265 | -0.0830683 | -0.0118227 | 0.0712455 | 4.93283E-06 |
| Plcb4 | 0.383702 | 0.255102 | 0.351721 | -0.128601 | -0.0319811 | 0.0966195 | 5.04649E-06 |
| B230209E15Rik | -2.87257 | -3.16592 | -2.60288 | -0.293351 | 0.26969 | 0.563041 | 5.54522E-06 |
| AK184713 | -1.35705 | 0.41657 | -1.43546 | 1.77362 | -0.0784054 | -1.85203 | 5.59725E-06 |
| Zfp423 | -0.457468 | 0.303199 | -0.51508 | 0.760667 | -0.057612 | -0.818279 | 7.19317E-06 |
| BC044745 | 1.81057 | 4.21084 | 2.71121 | 2.40027 | 0.900633 | -1.49963 | 7.66935E-06 |
| Psme4 | 0.333313 | 0.0831988 | 0.226553 | -0.250114 | -0.10676 | 0.143354 | 8.73543E-06 |
| Cyp11a1 | 1.4743 | -3.17024 | 1.57059 | -4.64454 | 0.0962909 | 4.74083 | 8.97957E-06 |
| Scn3a | -1.93472 | -2.28358 | -2.07755 | -0.348865 | -0.142826 | 0.206039 | 9.41073E-06 |
| Rasgrf2 | -2.26715 | -2.5682 | -2.0009 | -0.301042 | 0.266253 | 0.567295 | 9.74743E-06 |
| As3mt | 0.20733 | -0.156239 | 0.189649 | -0.363569 | -0.0176813 | 0.345888 | 1.04595E-05 |
| Plekhg3 | -0.183477 | 0.10999 | -0.165922 | 0.293467 | 0.0175558 | -0.275911 | 0.000011247 |
| Gcsh | 0.40301 | -0.407149 | 0.447008 | -0.810159 | 0.0439976 | 0.854156 | 1.39973E-05 |
| Spry4 | 0.800115 | -0.0795084 | 0.805547 | -0.879623 | 0.00543253 | 0.885056 | 1.49908E-05 |
| Copg2os2 | -0.444471 | -1.05463 | -0.578089 | -0.610159 | -0.133618 | 0.476541 | 1.55115E-05 |
| Rprm | -2.86855 | -2.84493 | -2.90839 | 0.0236186 | -0.0398388 | -0.0634574 | 1.70407E-05 |
| Trnp1 | 0.380325 | -0.174202 | 0.419196 | -0.554527 | 0.0388706 | 0.593397 | 1.76731E-05 |
| Asb11 | -1.31203 | -1.80545 | -1.33836 | -0.493419 | -0.026322 | 0.467097 | 1.77891E-05 |
| Rasd2 | -1.65059 | -2.00201 | -1.669 | -0.351419 | -0.0184189 | 0.333 | 1.78613E-05 |
| Chrm3 | -2.69898 | -3.06132 | -2.38186 | -0.362341 | 0.317124 | 0.679465 | 1.93143E-05 |
| Penk | -1.176 | -1.53588 | -1.12242 | -0.359877 | 0.05358 | 0.413457 | 2.07085E-05 |
| Mn1 | -2.16645 | -2.64428 | -2.01829 | -0.477827 | 0.148159 | 0.625986 | 0.000022585 |
| Prr7 | -2.54515 | -2.72562 | -2.56684 | -0.180478 | -0.0216903 | 0.158788 | 2.75302E-05 |
| Dync2h1 | 0.00819286 | -0.281213 | -0.214841 | -0.289406 | -0.223034 | 0.0663715 | 2.81173E-05 |
| Rasgrp1 | -1.44032 | -1.78872 | -1.57038 | -0.348399 | -0.130061 | 0.218338 | 3.47628E-05 |
| Sh3pxd2b | 0.617158 | -0.132266 | 0.477417 | -0.749424 | -0.139741 | 0.609683 | 3.51715E-05 |
| Svil | 0.208906 | -0.289781 | -0.0599291 | -0.498687 | -0.268835 | 0.229852 | 4.03743E-05 |
| Bcas1 | 0.111508 | -0.186698 | -0.0946746 | -0.298205 | -0.206182 | 0.0920231 | 4.10536E-05 |
| Hyou1 | 0.0127152 | 0.17687 | 0.212218 | 0.164154 | 0.199503 | 0.0353482 | 0.000044507 |
| Npy1r | -1.20931 | -1.52399 | -1.47405 | -0.314672 | -0.264735 | 0.0499367 | 4.50339E-05 |
| Cck | -0.714549 | -1.35651 | -0.613339 | -0.641965 | 0.101211 | 0.743175 | 5.05471E-05 |
| Ush1g | -1.31791 | 0.435796 | -1.35425 | 1.75371 | -0.0363434 | -1.79005 | 0.000052178 |
| Mzt1 | 0.298442 | -0.286804 | 0.393842 | -0.585247 | 0.0954001 | 0.680647 | 6.14179E-05 |
| Acaa1a | -0.59763 | 0.161396 | -0.734532 | 0.759026 | -0.136902 | -0.895929 | 6.70714E-05 |
| Pla2g7 | -0.421368 | 0.108047 | -0.401861 | 0.529415 | 0.0195069 | -0.509908 | 6.80393E-05 |
| 5830405F06Rik | -3.97878 | -3.83225 | -3.53718 | 0.146539 | 0.441609 | 0.29507 | 7.17771E-05 |
| Lrfn2 | -2.32981 | -3.13165 | -2.25194 | -0.801843 | 0.077865 | 0.879708 | 0.000077918 |
| Ldb2 | -2.41258 | -2.31109 | -2.16632 | 0.101487 | 0.246259 | 0.144772 | 7.97119E-05 |
| 8430408G22Rik | 0.521592 | -0.749564 | 0.997871 | -1.27116 | 0.476279 | 1.74743 | 7.97119E-05 |
| H2A | -1.6202 | 0.285302 | -1.58165 | 1.9055 | 0.0385465 | -1.86695 | 9.52757E-05 |
| Cckbr | -2.62124 | -2.51061 | -2.32793 | 0.11063 | 0.293301 | 0.182671 | 9.69656E-05 |
| Lzts1 | -2.67258 | -2.68662 | -2.73371 | -0.0140464 | -0.061131 | -0.0470846 | 0.000101653 |
| Elfn1 | -2.35167 | -2.77863 | -2.42036 | -0.426961 | -0.0686889 | 0.358272 | 0.000107848 |
| Cacng3 | -1.83978 | -2.23206 | -1.83894 | -0.392278 | 0.000847794 | 0.393126 | 0.000118893 |
| Foxo6 | -2.84659 | -2.95357 | -3.09124 | -0.106982 | -0.244654 | -0.137671 | 0.000119981 |
| St6galnac5 | -2.59675 | -2.328 | -2.54511 | 0.268753 | 0.0516437 | -0.21711 | 0.000120748 |
| Pttg1 | -2.00993 | -0.0353682 | -1.74638 | 1.97456 | 0.263544 | -1.71102 | 0.000129457 |
| Grem2 | -2.32034 | -2.58837 | -2.22168 | -0.268025 | 0.0986576 | 0.366683 | 0.000132813 |
| Stmn4 | -0.220754 | -0.605655 | -0.232685 | -0.384901 | -0.0119303 | 0.37297 | 0.000133741 |
| 4632415L05Rik | 1.00341 | 0.424932 | 1.0365 | -0.578479 | 0.0330904 | 0.611569 | 0.000135084 |
| Fzd7 | -0.131339 | 0.430755 | -0.0576863 | 0.562094 | 0.0736528 | -0.488442 | 0.000151422 |
| Zfp97 | 0.352368 | -0.839488 | 0.290231 | -1.19186 | -0.0621372 | 1.12972 | 0.000154913 |
| Cdon | -0.20551 | 0.272473 | -0.183726 | 0.477984 | 0.0217846 | -0.456199 | 0.000171639 |
| Jakmip1 | -0.772942 | -1.42358 | -0.911926 | -0.650639 | -0.138984 | 0.511655 | 0.000196916 |
| Hkdc1 | 1.13861 | -0.209829 | 0.595162 | -1.34844 | -0.543445 | 0.804991 | 0.000215601 |
| Gm1821 | -0.568576 | -0.0645705 | -0.575669 | 0.504006 | -0.00709269 | -0.511099 | 0.000215601 |
| Hpca | -1.75052 | -1.89331 | -1.79249 | -0.142792 | -0.0419768 | 0.100815 | 0.000221529 |
| St6gal2 | -1.47824 | -1.83605 | -1.09222 | -0.35781 | 0.38602 | 0.74383 | 0.000223106 |
| Nov | -1.44734 | -1.99933 | -1.86591 | -0.55199 | -0.418566 | 0.133424 | 0.000233672 |
| Mt3 | -0.575194 | -0.0456602 | -0.993284 | 0.529534 | -0.41809 | -0.947624 | 0.000253581 |
| AK047676 | -2.40736 | -2.49905 | -2.6634 | -0.0916906 | -0.256049 | -0.164358 | 0.000253807 |
| Myoc | -1.28532 | 0.762182 | -1.12391 | 2.0475 | 0.161416 | -1.88609 | 0.000285801 |
| Sass6 | 0.415344 | 0.142455 | 0.458609 | -0.272889 | 0.043265 | 0.316154 | 0.000327702 |
| Mfap2 | -1.1193 | 0.418458 | -0.663347 | 1.53776 | 0.455954 | -1.0818 | 0.000343593 |
| Cdc42ep2 | 0.764806 | 0.0245727 | 0.738092 | -0.740234 | -0.0267148 | 0.713519 | 0.000348496 |
| Zfp365 | -1.55339 | -1.79734 | -1.57037 | -0.24395 | -0.0169822 | 0.226968 | 0.000350444 |
| Npas2 | -2.16503 | -2.23962 | -2.04649 | -0.074589 | 0.11854 | 0.193129 | 0.000361791 |
| Creb3l1 | 1.39944 | 0.15039 | 1.5496 | -1.24905 | 0.150162 | 1.39921 | 0.000380663 |
| Prkar2b | -1.23201 | -1.6183 | -1.24982 | -0.386282 | -0.0178008 | 0.368482 | 0.0004588 |
| Ece1 | 0.295824 | 0.232497 | 0.196659 | -0.0633269 | -0.0991654 | -0.0358385 | 0.000463779 |
| Gabra3 | -0.96192 | -1.45694 | -0.969258 | -0.495015 | -0.0073378 | 0.487677 | 0.000472418 |
| Slc35f4 | 0.636753 | -0.2199 | 0.812382 | -0.856653 | 0.175629 | 1.03228 | 0.000472418 |
| Hba-a2 | 0.466348 | -0.14045 | 0.580076 | -0.606798 | 0.113727 | 0.720526 | 0.000472418 |
| Proca1 | -0.894787 | 0.25126 | -0.986716 | 1.14605 | -0.0919285 | -1.23798 | 0.000474637 |
| Col6a1 | -1.57409 | -1.77078 | -1.8876 | -0.19669 | -0.313509 | -0.116819 | 0.000503082 |
| Unc80 | 0.19148 | 0.104657 | 0.122725 | -0.086823 | -0.0687548 | 0.0180682 | 0.000506968 |
| Fndc9 | -2.2473 | -2.23955 | -2.13635 | 0.00775222 | 0.110953 | 0.103201 | 0.000560174 |
| Chst1 | -1.62622 | -1.44943 | -1.63964 | 0.176792 | -0.01342 | -0.190212 | 0.000637982 |
| Gm5083 | 1.12698 | 0.161048 | 1.03339 | -0.96593 | -0.0935875 | 0.872342 | 0.000651506 |
| Ifit2 | -0.921784 | 0.156787 | -1.01128 | 1.07857 | -0.0894944 | -1.16807 | 0.00065388 |
| Gsg1l | -1.50038 | -0.974661 | -1.68201 | 0.525719 | -0.181634 | -0.707353 | 0.000661583 |
| Evi2b | 0.390743 | -0.0831975 | 0.31997 | -0.47394 | -0.0707731 | 0.403167 | 0.000708779 |
| Pcp4l1 | -1.18735 | -1.44469 | -1.2384 | -0.257337 | -0.0510495 | 0.206287 | 0.000807581 |
| Crhr2 | -0.954312 | -1.31934 | -1.40904 | -0.365024 | -0.454731 | -0.0897076 | 0.000813419 |
| Egr1 | -1.65977 | -1.54382 | -1.86233 | 0.115949 | -0.202566 | -0.318515 | 0.000824694 |
| Tmem179 | -1.77733 | -1.87388 | -1.69324 | -0.0965538 | 0.084088 | 0.180642 | 0.000972091 |
| Mt2 | -0.60909 | -0.131802 | -1.05978 | 0.477288 | -0.450692 | -0.92798 | 0.000980735 |
| Thnsl1 | 0.249412 | -0.529023 | 0.24806 | -0.778435 | -0.0013519 | 0.777083 | 0.001024714 |
| Rragb | 0.998995 | 0.588315 | 1.00201 | -0.41068 | 0.00301536 | 0.413695 | 0.001045065 |
| Dock10 | -0.080549 | -0.438017 | -0.283372 | -0.357468 | -0.202823 | 0.154646 | 0.001045198 |
| Fas | 0.835909 | -1.03941 | 0.640197 | -1.87532 | -0.195712 | 1.67961 | 0.00105096 |
| Plk2 | -1.36151 | -1.58796 | -1.4488 | -0.226451 | -0.0872871 | 0.139164 | 0.00105096 |
| Hba-a2 | 0.509841 | 0.00216169 | 0.597963 | -0.507679 | 0.0881225 | 0.595801 | 0.00105096 |
| Nr4a1 | -0.0618999 | -0.655699 | -0.585983 | -0.593799 | -0.524083 | 0.0697161 | 0.00121944 |
| Sh3rf3 | -2.09854 | -2.15128 | -2.08127 | -0.0527424 | 0.0172709 | 0.0700133 | 0.001268646 |
| Neurod6 | -1.76386 | -2.14496 | -1.83302 | -0.381098 | -0.0691642 | 0.311934 | 0.001296005 |
| Nyap2 | -0.478798 | -0.184041 | -0.279121 | 0.294758 | 0.199677 | -0.0950808 | 0.001361133 |
| Dcc | -0.872393 | -1.74578 | -1.00101 | -0.87339 | -0.128617 | 0.744772 | 0.001372428 |
| Ncan | -1.70539 | -1.65994 | -1.66077 | 0.0454503 | 0.0446185 | -0.000831858 | 0.001396199 |
| Mboat2 | -0.532926 | -0.313213 | -0.499892 | 0.219713 | 0.0330341 | -0.186679 | 0.001546802 |
| Xlr3b | -0.426375 | 0.78898 | 0.0807149 | 1.21536 | 0.50709 | -0.708265 | 0.001590451 |
| Fam19a1 | -2.24741 | -2.62388 | -1.97714 | -0.376476 | 0.270268 | 0.646744 | 0.001603913 |
| Rnf207 | -1.62613 | -1.536 | -1.71546 | 0.0901307 | -0.089324 | -0.179455 | 0.001738275 |
| Calb2 | -0.26481 | 0.205986 | -0.174798 | 0.470796 | 0.0900116 | -0.380784 | 0.001741334 |
| 4933409K07Rik | -1.06461 | -0.375252 | -1.45469 | 0.689354 | -0.390083 | -1.07944 | 0.001793592 |
| Npas4 | 0.381545 | -0.478266 | -0.156004 | -0.859811 | -0.537549 | 0.322261 | 0.001798665 |
| Satb2 | -2.06182 | -2.39589 | -1.8803 | -0.334061 | 0.181523 | 0.515583 | 0.001816704 |
| Epha4 | -0.896853 | -1.28251 | -0.942813 | -0.385657 | -0.0459607 | 0.339697 | 0.001915449 |
| Fras1 | -1.53449 | -1.94717 | -1.67926 | -0.412679 | -0.144769 | 0.26791 | 0.001944165 |
| Rab27b | -1.04173 | -1.1626 | -0.892961 | -0.120871 | 0.14877 | 0.269641 | 0.002000058 |
| N6amt1 | 0.202786 | -0.255453 | -0.00301342 | -0.45824 | -0.2058 | 0.25244 | 0.002000058 |
| Htr2a | -1.83314 | -2.48362 | -1.86523 | -0.650484 | -0.0320867 | 0.618398 | 0.002065872 |
| 4933409K07Rik | -1.01213 | -0.349252 | -1.48658 | 0.662879 | -0.474448 | -1.13733 | 0.002099029 |
| Nde1 | 0.228257 | -0.0240852 | 0.23448 | -0.252343 | 0.00622253 | 0.258565 | 0.002361136 |
| Cx3cl1 | -1.54267 | -1.66521 | -1.41091 | -0.122541 | 0.131763 | 0.254304 | 0.002451548 |
| Neurog2 | -1.05699 | 0.610453 | -1.03229 | 1.66744 | 0.0247021 | -1.64274 | 0.002485164 |
| Fam189a1 | -0.940431 | -0.229189 | -0.966963 | 0.711242 | -0.0265325 | -0.737775 | 0.002561331 |
| Cacnb1 | -1.25991 | -1.13763 | -1.22938 | 0.122282 | 0.030527 | -0.0917553 | 0.002601491 |
| Ctnna3 | 1.13319 | 0.484547 | 1.23796 | -0.648645 | 0.104763 | 0.753408 | 0.002614212 |
| Dclk3 | -1.01688 | -1.47159 | -1.06 | -0.45471 | -0.043124 | 0.411586 | 0.002646947 |
| Oprd1 | -2.10141 | -2.48195 | -2.22048 | -0.380545 | -0.119078 | 0.261467 | 0.002673855 |
| Stap2 | -0.308543 | 0.342473 | -0.219701 | 0.651016 | 0.0888418 | -0.562175 | 0.002690751 |
| Tspan15 | 0.699619 | 0.247374 | 0.749181 | -0.452245 | 0.0495626 | 0.501808 | 0.002696667 |
| Fcrls | -0.408852 | -1.79533 | -0.554102 | -1.38648 | -0.14525 | 1.24123 | 0.002697923 |
| Ybx2 | -0.456338 | 0.144936 | -0.449719 | 0.601274 | 0.00661944 | -0.594654 | 0.002704317 |
| Tmem94 | -0.238515 | -0.124706 | -0.265635 | 0.113809 | -0.0271195 | -0.140929 | 0.002764002 |
| Hrk | -1.58839 | -1.25693 | -1.62208 | 0.331458 | -0.0336955 | -0.365154 | 0.002813144 |
| Ccdc122 | -1.81236 | -0.740299 | -1.9558 | 1.07206 | -0.143447 | -1.2155 | 0.002916542 |
| Egfem1 | -1.50518 | -1.65961 | -1.61788 | -0.154431 | -0.1127 | 0.0417315 | 0.002966376 |
| 1700086L19Rik | -2.47283 | -2.75649 | -2.63889 | -0.28366 | -0.166064 | 0.117596 | 0.003119672 |
| Kcne2 | 1.13975 | 0.177551 | 0.878967 | -0.962199 | -0.260784 | 0.701416 | 0.003226598 |
| Wfs1 | -0.925955 | -1.26238 | -0.998383 | -0.336428 | -0.0724282 | 0.264 | 0.003404501 |
| Pnma2 | -1.49096 | -1.74082 | -1.47742 | -0.249859 | 0.0135459 | 0.263405 | 0.003520189 |
| Rsph1 | 0.639449 | -0.452864 | 1.01833 | -1.09231 | 0.37888 | 1.47119 | 0.003543557 |
| Hist2h2be | 0.773524 | 0.0572828 | 0.78845 | -0.716241 | 0.0149262 | 0.731167 | 0.003543811 |
| Irf7 | -0.324286 | 0.49157 | 0.0501558 | 0.815856 | 0.374442 | -0.441415 | 0.003543811 |
| Hbb-bt | 0.484843 | -0.06926 | 0.502401 | -0.554103 | 0.017558 | 0.571661 | 0.003543811 |
| Mmp28 | -0.43082 | 0.106249 | -0.267958 | 0.537069 | 0.162862 | -0.374207 | 0.003543811 |
| Gng13 | 0.362912 | -0.0244792 | 0.392116 | -0.387391 | 0.0292039 | 0.416595 | 0.003543811 |
| Itga10 | 1.0245 | 0.311075 | 1.23774 | -0.713427 | 0.213239 | 0.926666 | 0.003589225 |
| Rcn1 | -1.15139 | -1.62363 | -1.19428 | -0.472242 | -0.0428826 | 0.429359 | 0.003694444 |
| Sh3bgr | -0.680898 | 0.455059 | -0.319755 | 1.13596 | 0.361142 | -0.774814 | 0.003699034 |
| Gpr15 | 1.08839 | -0.757269 | 0.771029 | -1.84566 | -0.317365 | 1.5283 | 0.003754417 |
| Myh7b | 0.893478 | -0.199031 | 0.302827 | -1.09251 | -0.590651 | 0.501857 | 0.003754417 |
| Osbpl3 | -0.0779552 | 0.13247 | -0.130217 | 0.210425 | -0.0522618 | -0.262687 | 0.003849141 |
| Cnih2 | -1.15124 | -1.2849 | -1.05971 | -0.133665 | 0.0915256 | 0.225191 | 0.00387614 |
| Hs3st2 | -2.14323 | -2.25033 | -1.88037 | -0.107102 | 0.26286 | 0.369961 | 0.003895787 |
| Gm14420 | 0.294466 | -0.295984 | 0.337554 | -0.590449 | 0.0430884 | 0.633538 | 0.003919302 |
| Fgd2 | -0.741426 | -0.883147 | -0.797483 | -0.14172 | -0.0560566 | 0.0856635 | 0.004240092 |
| Prkch | -1.75199 | -2.095 | -1.89481 | -0.343012 | -0.142827 | 0.200185 | 0.004277764 |
| Fhit | 0.0734447 | -0.789033 | 0.256765 | -0.862477 | 0.18332 | 1.0458 | 0.004317264 |
| AK134344 | 1.12901 | -0.0169281 | 0.792165 | -1.14594 | -0.336844 | 0.809093 | 0.00432592 |
| Sox11 | -1.00994 | -1.67603 | -0.992911 | -0.666087 | 0.0170281 | 0.683115 | 0.004375387 |
| Hbb-b1 | 0.497826 | 0.0419573 | 0.654251 | -0.455869 | 0.156425 | 0.612294 | 0.004761041 |
| Ngb | -2.55891 | -2.65379 | -1.59645 | -0.0948882 | 0.962458 | 1.05735 | 0.0052544 |
| Pkp4 | 0.222483 | 0.0985698 | 0.234695 | -0.123914 | 0.0122112 | 0.136125 | 0.0052544 |
| Galnt14 | -1.95457 | -2.44059 | -1.73225 | -0.486015 | 0.222319 | 0.708335 | 0.005623875 |
| Kctd16 | -2.08082 | -2.76238 | -2.11368 | -0.681554 | -0.0328558 | 0.648698 | 0.005948749 |
| Efcc1 | -1.63013 | -0.493856 | -1.80943 | 1.13627 | -0.179305 | -1.31558 | 0.005948749 |
| 1110051M20Rik | 0.0580225 | -0.432055 | 0.0461095 | -0.490078 | -0.011913 | 0.478165 | 0.005948749 |
| Nkain2 | -0.987691 | -1.34519 | -1.17645 | -0.357503 | -0.188758 | 0.168745 | 0.006120018 |
| Fam81a | -0.646263 | -1.08208 | -0.579585 | -0.435821 | 0.0666775 | 0.502498 | 0.006125423 |
| Begain | -1.01102 | -1.33172 | -0.977554 | -0.3207 | 0.0334636 | 0.354164 | 0.006149429 |
| Vipr1 | -1.56692 | -0.915997 | -1.35 | 0.650922 | 0.216917 | -0.434005 | 0.006362126 |
| Sgip1 | -0.525778 | 0.639902 | -0.897863 | 1.16568 | -0.372086 | -1.53777 | 0.006362126 |
| Hes5 | -2.40295 | -2.21393 | -2.39264 | 0.189018 | 0.0103047 | -0.178713 | 0.006379947 |
| Pld3 | -1.50526 | -1.56033 | -1.54696 | -0.0550726 | -0.0417032 | 0.0133694 | 0.006383439 |
| Col18a1 | 0.529651 | 0.121218 | 0.324205 | -0.408433 | -0.205446 | 0.202987 | 0.006478457 |
| A930006K02Rik | 1.49867 | -0.389406 | 1.19379 | -1.88808 | -0.304878 | 1.5832 | 0.006593989 |
| Tnc | 0.581757 | 0.204182 | 0.413792 | -0.377575 | -0.167966 | 0.209609 | 0.006772754 |
| 4933409K07Rik | -1.06433 | -0.387058 | -1.44245 | 0.677277 | -0.378113 | -1.05539 | 0.006855473 |
| Cdh13 | -1.46772 | -1.65014 | -1.42858 | -0.182417 | 0.039135 | 0.221552 | 0.006894863 |
| Cmip | -0.638938 | -0.748364 | -0.628052 | -0.109426 | 0.010886 | 0.120312 | 0.007040482 |
| Gm5796 | -0.532853 | 0.500012 | -0.594575 | 1.03286 | -0.0617222 | -1.09459 | 0.007047354 |
| Cecr6 | -1.52272 | -1.65695 | -1.74512 | -0.134231 | -0.222408 | -0.0881764 | 0.007065122 |
| Coch | -1.02779 | -1.17047 | -0.94483 | -0.142683 | 0.0829563 | 0.225639 | 0.008533248 |
| Serpini1 | 0.258118 | -0.160785 | 0.297569 | -0.418903 | 0.0394517 | 0.458354 | 0.008600345 |
| Plcb3 | 0.358594 | 0.00958917 | 0.249947 | -0.349005 | -0.108647 | 0.240358 | 0.008640518 |
| Mid1 | 2.84386 | -0.0902476 | -0.787284 | -2.93411 | -3.63115 | -0.697036 | 0.008796247 |
| Asb2 | -1.32321 | -1.22189 | -1.68755 | 0.101319 | -0.364343 | -0.465662 | 0.008991381 |
| Atp5o | 0.333593 | -0.110384 | 0.348982 | -0.443976 | 0.0153889 | 0.459365 | 0.009057958 |
| Slco1a4 | 0.173712 | -0.307613 | 0.0249762 | -0.481325 | -0.148736 | 0.332589 | 0.009057958 |
| AK041157 | -1.02748 | -0.380384 | -1.4797 | 0.647095 | -0.452219 | -1.09931 | 0.009306936 |
| Rnls | 0.685934 | -0.430316 | 0.587124 | -1.11625 | -0.0988099 | 1.01744 | 0.009306936 |
| 4933404O12Rik | 0.420041 | -0.426565 | 0.544306 | -0.846606 | 0.124265 | 0.970871 | 0.009627965 |
| Ndst4 | -2.01513 | -2.3468 | -1.275 | -0.331668 | 0.740133 | 1.0718 | 0.009655862 |
| Extl1 | -1.62182 | -1.70683 | -1.7168 | -0.0850112 | -0.0949859 | -0.00997469 | 0.010234403 |
| Vstm2a | -1.0181 | -1.31479 | -0.999034 | -0.296689 | 0.0190654 | 0.315755 | 0.010234403 |
| Slc1a1 | -0.575427 | -0.838153 | -0.633425 | -0.262727 | -0.0579987 | 0.204728 | 0.010430495 |
| Tubb2b | 0.362319 | -0.0489426 | 0.397994 | -0.411262 | 0.0356742 | 0.446936 | 0.010430495 |
| Sult2b1 | -1.11448 | -0.696454 | -1.07 | 0.418029 | 0.0444882 | -0.373541 | 0.010455084 |
| A330102I10Rik | -1.08742 | -1.13964 | -1.0865 | -0.0522185 | 0.000915909 | 0.0531344 | 0.010509947 |
| Eva1c | 0.644769 | 0.142337 | 0.59236 | -0.502432 | -0.0524085 | 0.450024 | 0.010677161 |
| Dkkl1 | -2.7956 | -3.08307 | -3.18328 | -0.287475 | -0.38768 | -0.100205 | 0.010737908 |
| Gpr26 | -1.84052 | -2.0713 | -1.52969 | -0.23078 | 0.310826 | 0.541606 | 0.01073856 |
| AK083197 | -1.10082 | -1.39988 | -1.03604 | -0.299063 | 0.0647765 | 0.363839 | 0.010786263 |
| Gm13152 | 1.89819 | 0.579091 | 1.35369 | -1.31909 | -0.544498 | 0.774596 | 0.010881051 |
| Kcnk1 | 0.499423 | 0.113957 | 0.560606 | -0.385466 | 0.0611839 | 0.44665 | 0.011280071 |
| Smim1 | 0.0976291 | -0.296626 | 0.283201 | -0.394255 | 0.185572 | 0.579827 | 0.011532288 |
| Banp | 0.121523 | -0.0105255 | 0.245216 | -0.132049 | 0.123692 | 0.255741 | 0.01201034 |
| Serpinb2 | -1.87841 | -0.200949 | -1.18802 | 1.67746 | 0.690393 | -0.987069 | 0.012057896 |
| Rasgef1c | -1.33768 | -0.590216 | -1.46332 | 0.747466 | -0.125642 | -0.873108 | 0.012409588 |
| AK030357 | -0.421404 | -2.03733 | -0.753652 | -1.61593 | -0.332248 | 1.28368 | 0.012678135 |
| Vipr2 | -0.264642 | 0.507436 | 0.0437832 | 0.772078 | 0.308425 | -0.463652 | 0.012816193 |
| Cplx3 | -1.89197 | -0.987942 | -1.66281 | 0.904026 | 0.229161 | -0.674865 | 0.012827292 |
| Ugt1a10 | -0.149355 | -0.951458 | -0.478444 | -0.802102 | -0.329089 | 0.473014 | 0.012881511 |
| Aldh1b1 | -2.12131 | -2.52562 | -2.22822 | -0.404312 | -0.106911 | 0.297401 | 0.013030044 |
| 4831440E17Rik | 0.540873 | -0.318793 | 0.373503 | -0.859666 | -0.16737 | 0.692296 | 0.01338973 |
| Gm8801 | 1.30658 | 0.138088 | 0.654359 | -1.16849 | -0.652223 | 0.516271 | 0.014047763 |
| Bspry | 0.469196 | -0.991859 | 0.871104 | -1.46105 | 0.401909 | 1.86296 | 0.014429877 |
| Dap | 0.862805 | -0.0186983 | 1.35701 | -0.881503 | 0.494205 | 1.37571 | 0.014641139 |
| Zdhhc23 | -1.21044 | -1.34051 | -1.01027 | -0.130071 | 0.200168 | 0.330239 | 0.01477507 |
| Tac2 | -3.22946 | -2.80128 | -3.21754 | 0.428179 | 0.0119182 | -0.416261 | 0.014813099 |
| Lrp1b | -0.160337 | -0.594443 | -0.232497 | -0.434105 | -0.0721602 | 0.361945 | 0.015050474 |
| Htr2c | -1.31117 | -1.43283 | -1.33301 | -0.121654 | -0.0218414 | 0.0998126 | 0.015939618 |
| Gstm6 | -0.563204 | -2.0012 | -0.498186 | -1.438 | 0.065018 | 1.50302 | 0.01636198 |
| Mok | -0.537119 | -0.289376 | -0.354166 | 0.247743 | 0.182953 | -0.0647894 | 0.016490435 |
| Snd1 | 0.0943319 | -0.344657 | -0.00653528 | -0.438989 | -0.100867 | 0.338122 | 0.016560752 |
| Fos | 0.914113 | 0.489357 | 0.815396 | -0.424756 | -0.0987168 | 0.326039 | 0.016717543 |
| Rimkla | 0.570672 | 0.0755699 | 0.600061 | -0.495102 | 0.0293887 | 0.524491 | 0.016989814 |
| Nrn1 | 0.523207 | 0.137941 | 0.243609 | -0.385266 | -0.279598 | 0.105668 | 0.018266882 |
| Npy2r | -2.39939 | -2.19924 | -2.00051 | 0.200148 | 0.398873 | 0.198725 | 0.018380797 |
| Sp7 | 0.612985 | -0.36087 | 0.497738 | -0.973855 | -0.115246 | 0.858609 | 0.019137232 |
| Spats1 | -1.00269 | -0.165431 | -0.797886 | 0.837257 | 0.204802 | -0.632455 | 0.019577242 |
| Hn1l | -0.691113 | 0.508391 | -0.622596 | 1.1995 | 0.0685177 | -1.13099 | 0.019669327 |
| Mmp17 | -1.15696 | -0.938878 | -0.946671 | 0.218082 | 0.210289 | -0.00779292 | 0.020335397 |
| Dazl | -1.9494 | -2.48097 | -2.10741 | -0.531569 | -0.158012 | 0.373558 | 0.02051268 |
| Vmn2r2 | -1.14652 | 0.0566221 | -1.36804 | 1.20314 | -0.221519 | -1.42466 | 0.02051268 |
| Nrsn1 | -1.06337 | -1.20333 | -0.869969 | -0.139962 | 0.193398 | 0.333361 | 0.02061699 |
| Actg1 | -0.236775 | -0.582957 | -0.327056 | -0.346182 | -0.0902818 | 0.255901 | 0.021219387 |
| 4933409K07Rik | -1.05946 | -0.407663 | -1.43495 | 0.651793 | -0.375492 | -1.02729 | 0.022269822 |
| Cacna1i | -0.606125 | -0.244234 | -0.541979 | 0.361891 | 0.0641454 | -0.297746 | 0.022627467 |
| Scd1 | -1.1671 | 0.769667 | -0.868072 | 1.93676 | 0.299024 | -1.63774 | 0.022798468 |
| Chmp1a | -0.566436 | -0.117414 | -0.55131 | 0.449022 | 0.015126 | -0.433896 | 0.022798468 |
| Slc30a3 | -1.66532 | -1.45855 | -1.70718 | 0.206763 | -0.0418655 | -0.248629 | 0.023127462 |
| Dcxr | -0.623424 | 0.188792 | -0.679241 | 0.812216 | -0.0558163 | -0.868033 | 0.023392762 |
| Traip | -1.92004 | -1.7303 | -1.93052 | 0.189741 | -0.0104784 | -0.200219 | 0.023522284 |
| E130012A19Rik | -1.5778 | -1.72882 | -1.32885 | -0.151018 | 0.248944 | 0.399962 | 0.023522284 |
| Slc7a3 | -0.841858 | -1.34597 | -0.663501 | -0.504107 | 0.178357 | 0.682465 | 0.023522284 |
| Rgs2 | -0.963444 | -1.21149 | -0.866427 | -0.248042 | 0.0970167 | 0.345059 | 0.023522284 |
| Pdk4 | 0.305106 | -0.384509 | 0.183732 | -0.689615 | -0.121374 | 0.568241 | 0.023693429 |
| Arhgdig | -1.72147 | -1.67754 | -1.44534 | 0.0439331 | 0.276135 | 0.232202 | 0.023988546 |
| Tifab | -0.158598 | -0.824725 | -0.453239 | -0.666127 | -0.294641 | 0.371486 | 0.024118216 |
| 5330417C22Rik | -1.75026 | -1.65768 | -2.03418 | 0.0925816 | -0.283921 | -0.376502 | 0.02434509 |
| Plppr4 | -0.769019 | -0.941738 | -0.922889 | -0.172719 | -0.153869 | 0.0188498 | 0.024407578 |
| Actg1 | -0.307318 | -0.754584 | -0.410389 | -0.447266 | -0.103071 | 0.344195 | 0.025360154 |
| Ubb | 0.308121 | -0.170829 | 0.395673 | -0.478951 | 0.0875513 | 0.566502 | 0.025360154 |
| Msln | -0.749011 | 0.314104 | -0.594051 | 1.06312 | 0.15496 | -0.908155 | 0.025448261 |
| Dgat2 | -1.40023 | -1.36963 | -1.4087 | 0.030599 | -0.00847411 | -0.0390731 | 0.02686227 |
| Nrarp | -1.62349 | -1.69103 | -1.46704 | -0.0675426 | 0.156451 | 0.223994 | 0.028227484 |
| Shprh | 0.0719077 | -0.0121573 | 0.0241531 | -0.0840651 | -0.0477546 | 0.0363105 | 0.028227484 |
| Sertm1 | -1.5184 | -1.9103 | -1.38904 | -0.391897 | 0.129358 | 0.521255 | 0.0288009 |
| Ppm1b | 0.199469 | 0.0618007 | 0.158289 | -0.137669 | -0.0411805 | 0.0964882 | 0.028967794 |
| Tmem132d | -1.57261 | -2.07364 | -1.90087 | -0.501033 | -0.328263 | 0.172771 | 0.030966025 |
| Rspo3 | 0.470241 | -0.429587 | 0.487445 | -0.899827 | 0.0172042 | 0.917032 | 0.031907005 |
| Klf16 | -1.45157 | -1.38147 | -1.52244 | 0.0701013 | -0.0708706 | -0.140972 | 0.031952906 |
| Necab3 | -0.574721 | -0.0835533 | -0.619843 | 0.491168 | -0.0451216 | -0.53629 | 0.032175819 |
| Vstm2b | -1.50067 | -1.82019 | -1.44824 | -0.31952 | 0.0524353 | 0.371955 | 0.032279172 |
| Gm15446 | 0.884919 | -0.341171 | 0.641107 | -1.22609 | -0.243812 | 0.982278 | 0.032308436 |
| Onecut2 | -0.730937 | 0.103292 | -0.631589 | 0.834229 | 0.0993486 | -0.73488 | 0.032663082 |
| Hhipl1 | -1.85179 | -0.791075 | -1.69006 | 1.06072 | 0.161733 | -0.898984 | 0.033039063 |
| Utp18 | -0.449577 | 0.0505442 | -0.505933 | 0.500121 | -0.0563567 | -0.556478 | 0.033903275 |
| Efcab14 | 0.193036 | -0.0200046 | 0.101028 | -0.21304 | -0.0920073 | 0.121033 | 0.033903275 |
| Kcnj2 | -1.55038 | -1.4869 | -1.57569 | 0.0634779 | -0.0253113 | -0.0887892 | 0.033957819 |
| Prkca | -0.176098 | -0.556251 | -0.174215 | -0.380153 | 0.00188255 | 0.382036 | 0.034468819 |
| Gm16432 | -0.792087 | -0.488274 | -0.646459 | 0.303812 | 0.145628 | -0.158184 | 0.034954713 |
| Gstt2 | -0.641882 | 0.156525 | -0.688428 | 0.798407 | -0.0465459 | -0.844953 | 0.035804396 |
| 2010204K13Rik | -2.27719 | -2.20036 | -2.08319 | 0.0768297 | 0.194003 | 0.117173 | 0.035980131 |
| Tox3 | -1.9879 | -2.17879 | -1.60625 | -0.190883 | 0.381652 | 0.572535 | 0.035980131 |
| Cyp46a1 | -1.13521 | -1.19505 | -1.12774 | -0.0598321 | 0.00747153 | 0.0673036 | 0.036308454 |
| Cyp4f15 | -1.38489 | -1.74582 | -1.24407 | -0.360926 | 0.140818 | 0.501745 | 0.037155192 |
| EG245297 | -1.53278 | -0.320622 | -1.51989 | 1.21216 | 0.0128905 | -1.19927 | 0.037155192 |
| Gm1943 | -0.334398 | 0.60827 | -0.346811 | 0.942668 | -0.0124131 | -0.955081 | 0.037155192 |
| Glra2 | -1.61346 | -1.76371 | -1.37446 | -0.150247 | 0.239005 | 0.389252 | 0.038373351 |
| Timp2 | -1.12072 | -1.05778 | -0.956915 | 0.0629391 | 0.163801 | 0.100862 | 0.038619827 |
| Limd2 | -0.758294 | -0.543228 | -0.792011 | 0.215066 | -0.0337174 | -0.248783 | 0.039927861 |
| Creg1 | 0.521909 | 0.189277 | 0.67385 | -0.332633 | 0.15194 | 0.484573 | 0.03997614 |
| AI593442 | -1.02185 | -1.13049 | -0.988373 | -0.108634 | 0.0334811 | 0.142115 | 0.040401317 |
| Zfp708 | -0.186568 | 0.128994 | -0.117074 | 0.315563 | 0.0694946 | -0.246068 | 0.041058482 |
| Xlr3c | -0.451057 | 0.879485 | 0.083909 | 1.33054 | 0.534966 | -0.795576 | 0.041962837 |
| AK080292 | 0.853052 | -0.0796647 | 1.11053 | -0.932717 | 0.257482 | 1.1902 | 0.042071892 |
| Plxnd1 | -1.03485 | -0.720179 | -1.11128 | 0.314671 | -0.0764264 | -0.391097 | 0.04215858 |
| Lgr4 | 0.531492 | 0.154775 | 0.463469 | -0.376717 | -0.0680236 | 0.308693 | 0.04215858 |
| Rerg | -1.35932 | -1.60378 | -1.2215 | -0.244468 | 0.137818 | 0.382286 | 0.042241971 |
| Cspg5 | -0.623454 | -0.684346 | -0.636265 | -0.0608921 | -0.0128107 | 0.0480814 | 0.042241971 |
| Tshz2 | 0.00371363 | -0.437752 | 0.2813 | -0.441465 | 0.277587 | 0.719052 | 0.042615405 |
| Gm3002 | -0.926883 | 0.257376 | -1.00109 | 1.18426 | -0.0742051 | -1.25846 | 0.042840601 |
| Eef1a1 | -0.216943 | -0.345176 | -0.219425 | -0.128233 | -0.00248254 | 0.125751 | 0.043119933 |
| Sdk2 | 0.0260761 | -0.585213 | -0.225576 | -0.61129 | -0.251652 | 0.359638 | 0.043963815 |
| Dhcr24 | 0.0979658 | -0.321111 | 0.1054 | -0.419077 | 0.00743442 | 0.426511 | 0.043996752 |
| Cd74 | -0.118161 | 0.61857 | -0.10817 | 0.736731 | 0.0099917 | -0.726739 | 0.044031865 |
| Spata18 | 0.453852 | 0.397292 | 0.661473 | -0.0565601 | 0.20762 | 0.26418 | 0.044440401 |
| Ddit4l | -1.76923 | -2.12354 | -1.84868 | -0.354316 | -0.0794477 | 0.274868 | 0.044451831 |
| Dlgap3 | -0.916564 | -0.810749 | -0.975018 | 0.105815 | -0.0584537 | -0.164269 | 0.044451831 |
| Pamr1 | -1.49865 | -0.933011 | -1.73993 | 0.565643 | -0.241277 | -0.80692 | 0.045694189 |
| Cnpy4 | 0.52373 | -0.0808413 | 0.605526 | -0.604571 | 0.0817966 | 0.686368 | 0.045813216 |
| Tmem51 | -0.310441 | 0.30353 | -0.108303 | 0.613971 | 0.202138 | -0.411833 | 0.045813216 |
| Crtac1 | -0.699432 | -1.25058 | -0.711507 | -0.551146 | -0.0120757 | 0.53907 | 0.04744247 |
| Camk2n1 | -1.06549 | -1.23014 | -1.04702 | -0.164654 | 0.0184697 | 0.183124 | 0.048270123 |
| 4933406I18Rik | -1.75626 | -0.221608 | -1.51914 | 1.53466 | 0.237128 | -1.29753 | 0.0494666 |
| Tmem68 | 0.27499 | -0.179846 | 0.321625 | -0.454836 | 0.0466355 | 0.501471 | 0.049617933 |
| Dpysl3 | -0.358386 | -0.48982 | -0.440416 | -0.131434 | -0.0820299 | 0.0494041 | 0.049634738 |

**Table B.** Enriched clusters of Gene Ontology (GO) biological process (BP), molecular function (MF) Functional Annotation Tool (FAT) categories, and KEGG pathways among the genes that exhibited significant genotype-by-environment interaction (FDR-adjusted P-value < 0.05) and differentially expressed (FDR-adjusted P-value < 0.05) between mice from genotype-by-environment groups in the three orthogonal contrasts (CB-HB, CF-HF, and HB-HF).

| Contrast and Category | Term | Count | P-value | FDR-adjusted P-value |
| --- | --- | --- | --- | --- |
| CB-HB |  |  |  |  |
| Annotation Cluster 1 | Enrichment Score: 3.163767954715416 |  |  |  |
| Category | Term | Count | P-value | FDR P-value |
| GOTERM_BP_FAT | GO:0030814~regulation of cAMP metabolic process | 8 | 4.77E-06 | 7.56E-03 |
| GOTERM_BP_FAT | GO:0030799~regulation of cyclic nucleotide metabolic process | 8 | 8.50E-06 | 1.35E-02 |
| GOTERM_BP_FAT | GO:0006140~regulation of nucleotide metabolic process | 8 | 1.02E-05 | 1.61E-02 |
| GOTERM_BP_FAT | GO:0045761~regulation of adenylate cyclase activity | 7 | 3.01E-05 | 4.78E-02 |
| GOTERM_BP_FAT | GO:0051339~regulation of lyase activity | 7 | 3.64E-05 | 5.77E-02 |
| GOTERM_BP_FAT | GO:0031279~regulation of cyclase activity | 7 | 3.64E-05 | 5.77E-02 |
| GOTERM_BP_FAT | GO:0007212~dopamine receptor signaling pathway | 5 | 4.23E-05 | 6.70E-02 |
| GOTERM_BP_FAT | GO:0030817~regulation of cAMP biosynthetic process | 7 | 4.77E-05 | 7.56E-02 |
| GOTERM_BP_FAT | GO:0030808~regulation of nucleotide biosynthetic process | 7 | 7.27E-05 | 1.15E-01 |
| GOTERM_BP_FAT | GO:0030802~regulation of cyclic nucleotide biosynthetic process | 7 | 7.27E-05 | 1.15E-01 |
| GOTERM_BP_FAT | GO:0007626~locomotory behavior | 11 | 1.53E-04 | 2.42E-01 |
| GOTERM_BP_FAT | GO:0019932~second-messenger-mediated signaling | 8 | 1.89E-04 | 2.99E-01 |
| GOTERM_BP_FAT | GO:0043085~positive regulation of catalytic activity | 11 | 3.11E-04 | 4.92E-01 |
| GOTERM_BP_FAT | GO:0007190~activation of adenylate cyclase activity | 5 | 7.50E-04 | 1.00E+00 |
| GOTERM_BP_FAT | GO:0051349~positive regulation of lyase activity | 5 | 8.22E-04 | 1.00E+00 |
| GOTERM_BP_FAT | GO:0045762~positive regulation of adenylate cyclase activity | 5 | 8.22E-04 | 1.00E+00 |
| GOTERM_BP_FAT | GO:0031281~positive regulation of cyclase activity | 5 | 8.22E-04 | 1.00E+00 |
| GOTERM_BP_FAT | GO:0044093~positive regulation of molecular function | 11 | 1.07E-03 | 1.00E+00 |
| GOTERM_BP_FAT | GO:0007188~G-protein signaling, coupled to cAMP nucleotide second messenger | 5 | 2.27E-03 | 1.00E+00 |
| GOTERM_BP_FAT | GO:0007187~G-protein signaling, coupled to cyclic nucleotide second messenger | 5 | 3.12E-03 | 1.00E+00 |
| GOTERM_BP_FAT | GO:0019933~cAMP-mediated signaling | 5 | 3.31E-03 | 1.00E+00 |
| GOTERM_BP_FAT | GO:0010579~positive regulation of adenylate cyclase activity by G-protein signaling pathway | 4 | 4.02E-03 | 1.00E+00 |
| GOTERM_BP_FAT | GO:0010578~regulation of adenylate cyclase activity involved in G-protein signaling | 4 | 4.02E-03 | 1.00E+00 |
| GOTERM_BP_FAT | GO:0007189~activation of adenylate cyclase activity by G-protein signaling pathway | 4 | 4.02E-03 | 1.00E+00 |
| GOTERM_BP_FAT | GO:0019935~cyclic-nucleotide-mediated signaling | 5 | 4.40E-03 | 1.00E+00 |
| GOTERM_BP_FAT | GO:0044092~negative regulation of molecular function | 6 | 1.08E-02 | 1.00E+00 |
| GOTERM_BP_FAT | GO:0007193~inhibition of adenylate cyclase activity by G-protein signaling | 3 | 1.24E-02 | 1.00E+00 |
| GOTERM_BP_FAT | GO:0051350~negative regulation of lyase activity | 3 | 1.70E-02 | 1.00E+00 |
| GOTERM_BP_FAT | GO:0007194~negative regulation of adenylate cyclase activity | 3 | 1.70E-02 | 1.00E+00 |
| GOTERM_BP_FAT | GO:0031280~negative regulation of cyclase activity | 3 | 1.70E-02 | 1.00E+00 |
| GOTERM_BP_FAT | GO:0050905~neuromuscular process | 4 | 2.26E-02 | 1.00E+00 |
| GOTERM_BP_FAT | GO:0043086~negative regulation of catalytic activity | 4 | 8.21E-02 | 1.00E+00 |
|  |  |  |  |  |
| Annotation Cluster 2 | Enrichment Score: 2.6207932325595507 |  |  |  |
| Category | Term | Count | PValue | FDR |
| KEGG_PATHWAY | mmu04080:Neuroactive ligand-receptor interaction | 15 | 3.60E-07 | 3.84E-04 |
| GOTERM_BP_FAT | GO:0007186~G-protein coupled receptor protein signaling pathway | 25 | 1.29E-01 | 1.00E+00 |
| GOTERM_BP_FAT | GO:0007166~cell surface receptor linked signal transduction | 29 | 2.96E-01 | 1.00E+00 |
|  |  |  |  |  |
| Annotation Cluster 3 | Enrichment Score: 2.0263750265168246 |  |  |  |
| Category | Term | Count | PValue | FDR |
| GOTERM_BP_FAT | GO:0051588~regulation of neurotransmitter transport | 5 | 6.28E-05 | 9.95E-02 |
| GOTERM_BP_FAT | GO:0019932~second-messenger-mediated signaling | 8 | 1.89E-04 | 2.99E-01 |
| GOTERM_BP_FAT | GO:0043085~positive regulation of catalytic activity | 11 | 3.11E-04 | 4.92E-01 |
| GOTERM_BP_FAT | GO:0050804~regulation of synaptic transmission | 7 | 5.16E-04 | 8.15E-01 |
| GOTERM_BP_FAT | GO:0051969~regulation of transmission of nerve impulse | 7 | 7.39E-04 | 1.00E+00 |
| KEGG_PATHWAY | mmu04020:Calcium signaling pathway | 9 | 8.44E-04 | 8.96E-01 |
| GOTERM_BP_FAT | GO:0031644~regulation of neurological system process | 7 | 9.84E-04 | 1.00E+00 |
| GOTERM_BP_FAT | GO:0051590~positive regulation of neurotransmitter transport | 3 | 9.89E-04 | 1.00E+00 |
| GOTERM_BP_FAT | GO:0046928~regulation of neurotransmitter secretion | 4 | 1.01E-03 | 1.00E+00 |
| GOTERM_BP_FAT | GO:0044093~positive regulation of molecular function | 11 | 1.07E-03 | 1.00E+00 |
| GOTERM_BP_FAT | GO:0007631~feeding behavior | 5 | 1.71E-03 | 1.00E+00 |
| GOTERM_BP_FAT | GO:0051952~regulation of amine transport | 4 | 1.96E-03 | 1.00E+00 |
| GOTERM_BP_FAT | GO:0007200~activation of phospholipase C activity by G-protein coupled receptor protein signaling pathway coupled to IP3 second messenger | 4 | 1.96E-03 | 1.00E+00 |
| GOTERM_BP_FAT | GO:0010518~positive regulation of phospholipase activity | 4 | 2.20E-03 | 1.00E+00 |
| GOTERM_BP_FAT | GO:0010863~positive regulation of phospholipase C activity | 4 | 2.20E-03 | 1.00E+00 |
| GOTERM_BP_FAT | GO:0007202~activation of phospholipase C activity | 4 | 2.20E-03 | 1.00E+00 |
| GOTERM_BP_FAT | GO:0060193~positive regulation of lipase activity | 4 | 2.46E-03 | 1.00E+00 |
| GOTERM_BP_FAT | GO:0051954~positive regulation of amine transport | 3 | 2.72E-03 | 1.00E+00 |
| GOTERM_BP_FAT | GO:0010517~regulation of phospholipase activity | 4 | 2.73E-03 | 1.00E+00 |
| GOTERM_BP_FAT | GO:0060191~regulation of lipase activity | 4 | 4.02E-03 | 1.00E+00 |
| GOTERM_BP_FAT | GO:0044057~regulation of system process | 8 | 4.26E-03 | 1.00E+00 |
| GOTERM_BP_FAT | GO:0051966~regulation of synaptic transmission, glutamatergic | 3 | 6.23E-03 | 1.00E+00 |
| GOTERM_BP_FAT | GO:0050433~regulation of catecholamine secretion | 3 | 6.23E-03 | 1.00E+00 |
| GOTERM_BP_FAT | GO:0048015~phosphoinositide-mediated signaling | 4 | 7.54E-03 | 1.00E+00 |
| GOTERM_BP_FAT | GO:0051046~regulation of secretion | 6 | 8.66E-03 | 1.00E+00 |
| GOTERM_BP_FAT | GO:0051345~positive regulation of hydrolase activity | 5 | 9.04E-03 | 1.00E+00 |
| KEGG_PATHWAY | mmu04540:Gap junction | 5 | 1.28E-02 | 1.00E+00 |
| GOTERM_MF_FAT | GO:0008227~amine receptor activity | 4 | 1.79E-02 | 1.00E+00 |
| GOTERM_BP_FAT | GO:0060341~regulation of cellular localization | 6 | 2.35E-02 | 1.00E+00 |
| GOTERM_BP_FAT | GO:0051240~positive regulation of multicellular organismal process | 6 | 2.46E-02 | 1.00E+00 |
| GOTERM_MF_FAT | GO:0043176~amine binding | 4 | 2.59E-02 | 1.00E+00 |
| GOTERM_BP_FAT | GO:0043279~response to alkaloid | 3 | 3.65E-02 | 1.00E+00 |
| GOTERM_BP_FAT | GO:0040013~negative regulation of locomotion | 3 | 3.88E-02 | 1.00E+00 |
| GOTERM_BP_FAT | GO:0051336~regulation of hydrolase activity | 6 | 4.83E-02 | 1.00E+00 |
| GOTERM_BP_FAT | GO:0014070~response to organic cyclic substance | 3 | 6.69E-02 | 1.00E+00 |
| GOTERM_BP_FAT | GO:0010959~regulation of metal ion transport | 3 | 8.43E-02 | 1.00E+00 |
| GOTERM_BP_FAT | GO:0048167~regulation of synaptic plasticity | 3 | 8.43E-02 | 1.00E+00 |
| GOTERM_BP_FAT | GO:0040012~regulation of locomotion | 4 | 9.98E-02 | 1.00E+00 |
| GOTERM_BP_FAT | GO:0051047~positive regulation of secretion | 3 | 9.98E-02 | 1.00E+00 |
| GOTERM_BP_FAT | GO:0043269~regulation of ion transport | 3 | 1.29E-01 | 1.00E+00 |
| GOTERM_BP_FAT | GO:0042493~response to drug | 3 | 2.22E-01 | 1.00E+00 |
| GOTERM_BP_FAT | GO:0051050~positive regulation of transport | 3 | 3.85E-01 | 1.00E+00 |
| GOTERM_BP_FAT | GO:0042127~regulation of cell proliferation | 7 | 4.60E-01 | 1.00E+00 |
| GOTERM_BP_FAT | GO:0042592~homeostatic process | 7 | 5.40E-01 | 1.00E+00 |
| GOTERM_BP_FAT | GO:0008284~positive regulation of cell proliferation | 4 | 5.49E-01 | 1.00E+00 |
| GOTERM_BP_FAT | GO:0010033~response to organic substance | 6 | 5.80E-01 | 1.00E+00 |
|  |  |  |  |  |
| Annotation Cluster 4 | Enrichment Score: 1.9927409056097374 |  |  |  |
| Category | Term | Count | PValue | FDR |
| GOTERM_MF_FAT | GO:0042165~neurotransmitter binding | 6 | 2.17E-03 | 1.00E+00 |
| GOTERM_MF_FAT | GO:0030594~neurotransmitter receptor activity | 6 | 2.17E-03 | 1.00E+00 |
| GOTERM_MF_FAT | GO:0004983~neuropeptide Y receptor activity | 3 | 6.69E-03 | 1.00E+00 |
| GOTERM_MF_FAT | GO:0042923~neuropeptide binding | 4 | 7.23E-03 | 1.00E+00 |
| GOTERM_MF_FAT | GO:0008188~neuropeptide receptor activity | 4 | 7.23E-03 | 1.00E+00 |
| GOTERM_MF_FAT | GO:0001653~peptide receptor activity | 5 | 2.83E-02 | 1.00E+00 |
| GOTERM_MF_FAT | GO:0008528~peptide receptor activity, G-protein coupled | 5 | 2.83E-02 | 1.00E+00 |
| GOTERM_MF_FAT | GO:0042277~peptide binding | 5 | 8.71E-02 | 1.00E+00 |
|  |  |  |  |  |
| Annotation Cluster 5 | Enrichment Score: 1.6622966571882283 |  |  |  |
| Category | Term | Count | PValue | FDR |
| GOTERM_BP_FAT | GO:0007610~behavior | 16 | 1.42E-05 | 2.24E-02 |
| GOTERM_BP_FAT | GO:0050877~neurological system process | 16 | 7.32E-01 | 1.00E+00 |
| GOTERM_BP_FAT | GO:0050890~cognition | 8 | 9.94E-01 | 1.00E+00 |
|  |  |  |  |  |
| Annotation Cluster 6 | Enrichment Score: 1.583637106135891 |  |  |  |
| Category | Term | Count | PValue | FDR |
| GOTERM_BP_FAT | GO:0007631~feeding behavior | 5 | 1.71E-03 | 1.00E+00 |
| GOTERM_BP_FAT | GO:0003013~circulatory system process | 4 | 1.02E-01 | 1.00E+00 |
| GOTERM_BP_FAT | GO:0008015~blood circulation | 4 | 1.02E-01 | 1.00E+00 |
|  |  |  |  |  |
| Annotation Cluster 7 | Enrichment Score: 1.3429710136241038 |  |  |  |
| Category | Term | Count | PValue | FDR |
| GOTERM_MF_FAT | GO:0005509~calcium ion binding | 22 | 1.67E-04 | 2.23E-01 |
| GOTERM_MF_FAT | GO:0043167~ion binding | 52 | 4.23E-02 | 1.00E+00 |
| GOTERM_MF_FAT | GO:0046872~metal ion binding | 51 | 4.35E-02 | 1.00E+00 |
| GOTERM_MF_FAT | GO:0043169~cation binding | 51 | 5.03E-02 | 1.00E+00 |
| GOTERM_MF_FAT | GO:0008270~zinc ion binding | 21 | 7.16E-01 | 1.00E+00 |
| GOTERM_MF_FAT | GO:0046914~transition metal ion binding | 25 | 7.89E-01 | 1.00E+00 |
|  |  |  |  |  |
| Annotation Cluster 8 | Enrichment Score: 1.3353514418463923 |  |  |  |
| Category | Term | Count | PValue | FDR |
| GOTERM_MF_FAT | GO:0022843~voltage-gated cation channel activity | 7 | 2.25E-03 | 1.00E+00 |
| GOTERM_MF_FAT | GO:0022832~voltage-gated channel activity | 8 | 2.75E-03 | 1.00E+00 |
| GOTERM_MF_FAT | GO:0005244~voltage-gated ion channel activity | 8 | 2.75E-03 | 1.00E+00 |
| GOTERM_MF_FAT | GO:0022836~gated channel activity | 9 | 9.41E-03 | 1.00E+00 |
| GOTERM_MF_FAT | GO:0005261~cation channel activity | 8 | 1.54E-02 | 1.00E+00 |
| GOTERM_MF_FAT | GO:0005249~voltage-gated potassium channel activity | 5 | 2.01E-02 | 1.00E+00 |
| GOTERM_MF_FAT | GO:0005216~ion channel activity | 9 | 3.03E-02 | 1.00E+00 |
| GOTERM_MF_FAT | GO:0046873~metal ion transmembrane transporter activity | 8 | 3.27E-02 | 1.00E+00 |
| GOTERM_BP_FAT | GO:0030001~metal ion transport | 10 | 3.49E-02 | 1.00E+00 |
| GOTERM_MF_FAT | GO:0030955~potassium ion binding | 5 | 3.53E-02 | 1.00E+00 |
| GOTERM_MF_FAT | GO:0022838~substrate specific channel activity | 9 | 3.54E-02 | 1.00E+00 |
| GOTERM_MF_FAT | GO:0015267~channel activity | 9 | 3.79E-02 | 1.00E+00 |
| GOTERM_MF_FAT | GO:0022803~passive transmembrane transporter activity | 9 | 3.79E-02 | 1.00E+00 |
| GOTERM_MF_FAT | GO:0005267~potassium channel activity | 5 | 4.64E-02 | 1.00E+00 |
| GOTERM_BP_FAT | GO:0006812~cation transport | 10 | 7.64E-02 | 1.00E+00 |
| GOTERM_BP_FAT | GO:0006813~potassium ion transport | 5 | 7.85E-02 | 1.00E+00 |
| GOTERM_BP_FAT | GO:0006816~calcium ion transport | 4 | 1.23E-01 | 1.00E+00 |
| GOTERM_MF_FAT | GO:0005262~calcium channel activity | 3 | 1.59E-01 | 1.00E+00 |
| GOTERM_MF_FAT | GO:0031420~alkali metal ion binding | 5 | 1.70E-01 | 1.00E+00 |
| GOTERM_BP_FAT | GO:0006811~ion transport | 11 | 1.82E-01 | 1.00E+00 |
| GOTERM_BP_FAT | GO:0015674~di-, tri-valent inorganic cation transport | 4 | 2.22E-01 | 1.00E+00 |
| KEGG_PATHWAY | mmu04010:MAPK signaling pathway | 5 | 3.11E-01 | 1.00E+00 |
| GOTERM_BP_FAT | GO:0015672~monovalent inorganic cation transport | 5 | 3.65E-01 | 1.00E+00 |
| GOTERM_BP_FAT | GO:0055085~transmembrane transport | 5 | 6.86E-01 | 1.00E+00 |
|  |  |  |  |  |
| Annotation Cluster 9 | Enrichment Score: 1.3324501841238363 |  |  |  |
| Category | Term | Count | PValue | FDR |
| GOTERM_BP_FAT | GO:0019226~transmission of nerve impulse | 7 | 2.69E-02 | 1.00E+00 |
| GOTERM_BP_FAT | GO:0007267~cell-cell signaling | 8 | 2.76E-02 | 1.00E+00 |
| GOTERM_BP_FAT | GO:0007268~synaptic transmission | 6 | 3.42E-02 | 1.00E+00 |
| GOTERM_BP_FAT | GO:0046903~secretion | 5 | 1.84E-01 | 1.00E+00 |
|  |  |  |  |  |
| Annotation Cluster 10 | Enrichment Score: 1.174041710414672 |  |  |  |
| Category | Term | Count | PValue | FDR |
| GOTERM_BP_FAT | GO:0007155~cell adhesion | 11 | 5.73E-02 | 1.00E+00 |
| GOTERM_BP_FAT | GO:0022610~biological adhesion | 11 | 5.79E-02 | 1.00E+00 |
| GOTERM_BP_FAT | GO:0016337~cell-cell adhesion | 6 | 9.05E-02 | 1.00E+00 |
|  |  |  |  |  |
| Annotation Cluster 11 | Enrichment Score: 0.9515180976124333 |  |  |  |
| Category | Term | Count | PValue | FDR |
| GOTERM_BP_FAT | GO:0007611~learning or memory | 5 | 1.25E-02 | 1.00E+00 |
| GOTERM_BP_FAT | GO:0007612~learning | 3 | 1.13E-01 | 1.00E+00 |
| GOTERM_BP_FAT | GO:0050890~cognition | 8 | 9.94E-01 | 1.00E+00 |
|  |  |  |  |  |
| Contrast and Category | Term | Count | P-value | FDR-P-value |
| CF-HF |  |  |  |  |
| Annotation Cluster 1 | Enrichment Score: 1.8625810706749328 |  |  |  |
| Category | Term | Count | PValue | FDR |
| GOTERM_BP_FAT | GO:0015669~gas transport | 4 | 3.21E-04 | 5.10E-01 |
| GOTERM_MF_FAT | GO:0005344~oxygen transporter activity | 3 | 5.85E-03 | 7.62E+00 |
| GOTERM_BP_FAT | GO:0015671~oxygen transport | 3 | 5.92E-03 | 9.02E+00 |
| GOTERM_MF_FAT | GO:0019825~oxygen binding | 3 | 9.49E-03 | 1.21E+01 |
| GOTERM_MF_FAT | GO:0020037~heme binding | 5 | 6.84E-02 | 6.16E+01 |
| GOTERM_MF_FAT | GO:0046906~tetrapyrrole binding | 5 | 7.86E-02 | 6.69E+01 |
| GOTERM_MF_FAT | GO:0005506~iron ion binding | 7 | 1.61E-01 | 9.07E+01 |
|  |  |  |  |  |
| Annotation Cluster 2 | Enrichment Score: 1.3207382222772561 |  |  |  |
| Category | Term | Count | PValue | FDR |
| KEGG_PATHWAY | mmu03320:PPAR signaling pathway | 5 | 1.01E-02 | 1.04E+01 |
| KEGG_PATHWAY | mmu01040:Biosynthesis of unsaturated fatty acids | 3 | 3.39E-02 | 3.11E+01 |
| GOTERM_BP_FAT | GO:0006631~fatty acid metabolic process | 4 | 3.17E-01 | 9.98E+01 |
|  |  |  |  |  |
| Annotation Cluster 3 | Enrichment Score: 1.1979310087835378 |  |  |  |
| Category | Term | Count | PValue | FDR |
| GOTERM_BP_FAT | GO:0040012~regulation of locomotion | 5 | 3.08E-02 | 3.92E+01 |
| GOTERM_BP_FAT | GO:0030334~regulation of cell migration | 4 | 7.66E-02 | 7.19E+01 |
| GOTERM_BP_FAT | GO:0051270~regulation of cell motion | 4 | 1.08E-01 | 8.38E+01 |
|  |  |  |  |  |
| Annotation Cluster 4 | Enrichment Score: 1.146285709906526 |  |  |  |
| Category | Term | Count | PValue | FDR |
| KEGG_PATHWAY | mmu04916:Melanogenesis | 5 | 2.24E-02 | 2.17E+01 |
| KEGG_PATHWAY | mmu04310:Wnt signaling pathway | 5 | 7.66E-02 | 5.76E+01 |
| KEGG_PATHWAY | mmu04912:GnRH signaling pathway | 4 | 8.68E-02 | 6.24E+01 |
| KEGG_PATHWAY | mmu04720:Long-term potentiation | 3 | 1.75E-01 | 8.74E+01 |
|  |  |  |  |  |
| Annotation Cluster 5 | Enrichment Score: 1.0717453709291391 |  |  |  |
| Category | Term | Count | PValue | FDR |
| GOTERM_BP_FAT | GO:0015669~gas transport | 4 | 3.21E-04 | 5.10E-01 |
| GOTERM_BP_FAT | GO:0030097~hemopoiesis | 5 | 2.84E-01 | 9.95E+01 |
| GOTERM_BP_FAT | GO:0048872~homeostasis of number of cells | 3 | 3.43E-01 | 9.99E+01 |
| GOTERM_BP_FAT | GO:0048534~hemopoietic or lymphoid organ development | 5 | 3.57E-01 | 9.99E+01 |
| GOTERM_BP_FAT | GO:0002520~immune system development | 5 | 3.91E-01 | 1.00E+02 |
|  |  |  |  |  |
| Annotation Cluster 6 | Enrichment Score: 0.9264113261842972 |  |  |  |
| Category | Term | Count | PValue | FDR |
| KEGG_PATHWAY | mmu04540:Gap junction | 4 | 6.54E-02 | 5.18E+01 |
| KEGG_PATHWAY | mmu04270:Vascular smooth muscle contraction | 4 | 1.39E-01 | 8.02E+01 |
| KEGG_PATHWAY | mmu04730:Long-term depression | 3 | 1.82E-01 | 8.86E+01 |
|  |  |  |  |  |
| Annotation Cluster 7 | Enrichment Score: 0.9182926828014395 |  |  |  |
| Category | Term | Count | PValue | FDR |
| GOTERM_BP_FAT | GO:0016053~organic acid biosynthetic process | 5 | 6.55E-02 | 6.60E+01 |
| GOTERM_BP_FAT | GO:0046394~carboxylic acid biosynthetic process | 5 | 6.55E-02 | 6.60E+01 |
| GOTERM_BP_FAT | GO:0008610~lipid biosynthetic process | 7 | 8.71E-02 | 7.66E+01 |
| GOTERM_BP_FAT | GO:0006633~fatty acid biosynthetic process | 3 | 2.16E-01 | 9.79E+01 |
| GOTERM_BP_FAT | GO:0006631~fatty acid metabolic process | 4 | 3.17E-01 | 9.98E+01 |
|  |  |  |  |  |
| Contrast and Category | Term | Count | P-value | FDR-P-value |
| HB-HF |  |  |  |  |
| Annotation Cluster 1 | Enrichment Score: 0.944848442208523 |  |  |  |
| Category | Term | Count | PValue | FDR |
| GOTERM_MF_FAT | GO:0008270~zinc ion binding | 6 | 7.50E-02 | 1.00E+00 |
| GOTERM_MF_FAT | GO:0046872~metal ion binding | 8 | 1.12E-01 | 1.00E+00 |
| GOTERM_MF_FAT | GO:0043169~cation binding | 8 | 1.17E-01 | 1.00E+00 |
| GOTERM_MF_FAT | GO:0043167~ion binding | 8 | 1.24E-01 | 1.00E+00 |
| GOTERM_MF_FAT | GO:0046914~transition metal ion binding | 6 | 1.55E-01 | 1.00E+00 |
|  |  |  |  |  |
| Annotation Cluster 2 | Enrichment Score: 0.9031701769465759 |  |  |  |
| Category | Term | Count | PValue | FDR |
| GOTERM_BP_FAT | GO:0006350~transcription | 7 | 1.68E-02 | 1.00E+00 |
| GOTERM_BP_FAT | GO:0045449~regulation of transcription | 7 | 4.68E-02 | 1.00E+00 |
| GOTERM_BP_FAT | GO:0045893~positive regulation of transcription, DNA-dependent | 3 | 9.40E-02 | 1.00E+00 |
| GOTERM_BP_FAT | GO:0051254~positive regulation of RNA metabolic process | 3 | 9.52E-02 | 1.00E+00 |
| GOTERM_BP_FAT | GO:0045941~positive regulation of transcription | 3 | 1.17E-01 | 1.00E+00 |
| GOTERM_BP_FAT | GO:0010628~positive regulation of gene expression | 3 | 1.23E-01 | 1.00E+00 |
| GOTERM_BP_FAT | GO:0045935~positive regulation of nucleobase, nucleoside, nucleotide and nucleic acid metabolic process | 3 | 1.32E-01 | 1.00E+00 |
| GOTERM_BP_FAT | GO:0051173~positive regulation of nitrogen compound metabolic process | 3 | 1.39E-01 | 1.00E+00 |
| GOTERM_BP_FAT | GO:0010557~positive regulation of macromolecule biosynthetic process | 3 | 1.41E-01 | 1.00E+00 |
| GOTERM_BP_FAT | GO:0031328~positive regulation of cellular biosynthetic process | 3 | 1.50E-01 | 1.00E+00 |
| GOTERM_BP_FAT | GO:0009891~positive regulation of biosynthetic process | 3 | 1.52E-01 | 1.00E+00 |
| GOTERM_BP_FAT | GO:0010604~positive regulation of macromolecule metabolic process | 3 | 1.86E-01 | 1.00E+00 |
| GOTERM_BP_FAT | GO:0006355~regulation of transcription, DNA-dependent | 4 | 2.75E-01 | 1.00E+00 |
| GOTERM_BP_FAT | GO:0051252~regulation of RNA metabolic process | 4 | 2.83E-01 | 1.00E+00 |
| GOTERM_MF_FAT | GO:0003677~DNA binding | 4 | 3.26E-01 | 1.00E+00 |

**Table C.** Genes differentially expressed (FDR-adjusted P-value < 0.05) between mice from the Control and High activity genotypes.

| **Gene Symbol** | **Log2(Control//High activity genotype)** | **FDR-adjusted P-value** |
| --- | --- | --- |
| 1700086L19Rik | -2.06993 | 0.0064553 |
| 3110035E14Rik | -1.6564 | 0.0064553 |
| 4632415L05Rik | 0.774436 | 0.0064553 |
| 6330403A02Rik | -1.90049 | 0.0064553 |
| Actn2 | -3.34287 | 0.0064553 |
| Adcy5 | -1.46634 | 0.0064553 |
| Adi1 | 0.960572 | 0.0064553 |
| Adora2a | -3.75852 | 0.0064553 |
| AK036371 | 1.37381 | 0.0064553 |
| AK040671 | 4.26887 | 0.0064553 |
| AK047676 | -2.01973 | 0.0064553 |
| AK133650 | -2.43712 | 0.0064553 |
| AK184713 | -1.88134 | 0.0064553 |
| Akap5 | -2.13403 | 0.0064553 |
| Aldh1a1 | 0.807717 | 0.0064553 |
| Ankrd63 | -4.39899 | 0.0064553 |
| Arl5a | 0.631508 | 0.0064553 |
| Asic4 | -2.58254 | 0.0064553 |
| Basp1 | -2.16316 | 0.0064553 |
| C130074G19Rik | -0.891012 | 0.0064553 |
| Cacna1g | 1.69087 | 0.0064553 |
| Cacna1h | -2.02555 | 0.0064553 |
| Camk2a | -2.01713 | 0.0064553 |
| Ccl28 | -2.88918 | 0.0064553 |
| Cdh9 | -2.22645 | 0.0064553 |
| Chn1 | -0.964636 | 0.0064553 |
| Chrm1 | -4.19663 | 0.0064553 |
| Chrm3 | -1.92345 | 0.0064553 |
| Chst1 | -1.25179 | 0.0064553 |
| Cnih3 | -1.90971 | 0.0064553 |
| Col6a1 | -1.28623 | 0.0064553 |
| Cpne5 | -4.47141 | 0.0064553 |
| Cpne6 | -1.73018 | 0.0064553 |
| Creb3l1 | 1.40607 | 0.0064553 |
| Creg2 | -1.5764 | 0.0064553 |
| Crhbp | -3.56026 | 0.0064553 |
| Cyp46a1 | -0.791861 | 0.0064553 |
| D430019H16Rik | -3.0582 | 0.0064553 |
| Dbpht2 | -2.42269 | 0.0064553 |
| Dlk1 | -1.89208 | 0.0064553 |
| Dlx1 | -4.80199 | 0.0064553 |
| Dlx2 | -3.93618 | 0.0064553 |
| Drd1a | -4.57751 | 0.0064553 |
| Egr1 | -1.38406 | 0.0064553 |
| Enc1 | -2.59375 | 0.0064553 |
| Fam124a | -1.47648 | 0.0064553 |
| Fam163b | -1.55373 | 0.0064553 |
| Fibcd1 | -2.38026 | 0.0064553 |
| Foxg1 | -5.19633 | 0.0064553 |
| Gda | -4.30171 | 0.0064553 |
| Gm14204 | -1.03369 | 0.0064553 |
| Gm1821 | -0.624371 | 0.0064553 |
| Gng4 | -2.52811 | 0.0064553 |
| Gng7 | -2.57581 | 0.0064553 |
| Gpd2 | 0.848664 | 0.0064553 |
| Gpr6 | -4.71176 | 0.0064553 |
| Grasp | -1.76297 | 0.0064553 |
| Grem2 | -1.70727 | 0.0064553 |
| Hebp2 | 1.46019 | 0.0064553 |
| Htr1a | -2.49493 | 0.0064553 |
| Kcna4 | -1.56449 | 0.0064553 |
| Kcnf1 | -4.26674 | 0.0064553 |
| Kcng1 | -4.01258 | 0.0064553 |
| Kcnh3 | -2.29195 | 0.0064553 |
| Kcnh4 | -3.18327 | 0.0064553 |
| Kcnj4 | -4.42276 | 0.0064553 |
| Kcnv1 | -4.88982 | 0.0064553 |
| Kif19a | 1.56706 | 0.0064553 |
| Klf16 | -1.13552 | 0.0064553 |
| Krt25 | -1.78723 | 0.0064553 |
| Lamp5 | -2.6147 | 0.0064553 |
| Lipg | -2.94956 | 0.0064553 |
| Lrrc10b | -4.28336 | 0.0064553 |
| Mal2 | -2.10233 | 0.0064553 |
| Mchr1 | -2.97991 | 0.0064553 |
| Meis2 | -2.63925 | 0.0064553 |
| Meis2 | -2.45995 | 0.0064553 |
| Mpped1 | -2.44776 | 0.0064553 |
| Myo15 | -1.82505 | 0.0064553 |
| Ncald | -1.47623 | 0.0064553 |
| Neto1 | -2.64553 | 0.0064553 |
| Neurl1b | -3.16774 | 0.0064553 |
| Ngef | -2.50917 | 0.0064553 |
| Npas2 | -1.62593 | 0.0064553 |
| Nsg2 | -1.59426 | 0.0064553 |
| Nt5dc3 | 1.7349 | 0.0064553 |
| Nts | -2.72781 | 0.0064553 |
| Pak6 | -2.9217 | 0.0064553 |
| Plk2 | -1.0066 | 0.0064553 |
| Prr7 | -2.02265 | 0.0064553 |
| Prss12 | -3.04173 | 0.0064553 |
| Ptgs2 | -1.60193 | 0.0064553 |
| Ptprv | -3.31905 | 0.0064553 |
| Rab40b | -2.59439 | 0.0064553 |
| Rapgefl1 | -1.00723 | 0.0064553 |
| Rasgef1c | -1.24746 | 0.0064553 |
| Rasgrf2 | -1.55026 | 0.0064553 |
| Rgl1 | -0.718169 | 0.0064553 |
| Rgs4 | -2.00756 | 0.0064553 |
| Rin1 | -2.53729 | 0.0064553 |
| Rprm | -2.34433 | 0.0064553 |
| Rprml | -3.60267 | 0.0064553 |
| Rtn4rl2 | -3.45839 | 0.0064553 |
| Rxrg | -3.8042 | 0.0064553 |
| Scn3a | -1.83386 | 0.0064553 |
| Sh3rf3 | -1.58063 | 0.0064553 |
| Slc35d3 | -2.85678 | 0.0064553 |
| Slit1 | -2.51886 | 0.0064553 |
| Slu7 | -2.1905 | 0.0064553 |
| Snhg11 | -0.98229 | 0.0064553 |
| Sostdc1 | 2.1901 | 0.0064553 |
| Sowaha | -0.736885 | 0.0064553 |
| Sp9 | -4.80959 | 0.0064553 |
| Spry4 | 0.796085 | 0.0064553 |
| Sst | -3.11108 | 0.0064553 |
| Stom | 1.10491 | 0.0064553 |
| Syndig1l | -1.63453 | 0.0064553 |
| Syt16 | -1.78874 | 0.0064553 |
| Tbr1 | -3.7204 | 0.0064553 |
| Tmem179 | -1.2966 | 0.0064553 |
| Tpm3 | 1.33002 | 0.0064553 |
| Tubb2b | 0.564081 | 0.0064553 |
| Vdr | -3.4123 | 0.0064553 |
| Zfp365 | -1.1554 | 0.0064553 |
| Zfp831 | -4.47846 | 0.0064553 |
| 2010300C02Rik | -1.55499 | 0.0119217 |
| 4930539E08Rik | -2.46176 | 0.0119217 |
| Agap2 | -0.869476 | 0.0119217 |
| Mcm6 | -1.83045 | 0.0119217 |
| Myo5b | -1.89566 | 0.0119217 |
| Nov | -1.12766 | 0.0119217 |
| Prss56 | -5.79256 | 0.0119217 |
| Slc22a3 | -3.06188 | 0.0119217 |
| Thbs4 | -2.26092 | 0.0119217 |
| Timp2 | -0.686978 | 0.0119217 |
| Ubb | 0.724932 | 0.0119217 |
| Alcam | -0.629497 | 0.0163518 |
| Arhgdig | -1.14968 | 0.0163518 |
| Chsy3 | -1.91777 | 0.0163518 |
| Htr1f | -3.1908 | 0.0163518 |
| Ifit2 | -1.02526 | 0.0163518 |
| Kcnj2 | -1.18881 | 0.0163518 |
| Krt9 | -3.65146 | 0.0163518 |
| Lrfn2 | -1.68567 | 0.0163518 |
| Pcdh10 | -0.928496 | 0.0163518 |
| Pkd2l1 | -1.82768 | 0.0163518 |
| Sstr4 | -2.52344 | 0.0163518 |
| Cdc42ep2 | 0.742809 | 0.0207526 |
| Dap | 1.14772 | 0.0207526 |
| Pamr1 | -1.41168 | 0.0207526 |
| Prkch | -1.35524 | 0.0207526 |
| Synpo | -1.94253 | 0.0207526 |
| Creg1 | 0.525841 | 0.0251039 |
| Crh | -2.87987 | 0.0251039 |
| Pde10a | -2.7352 | 0.0251039 |
| Slc17a8 | -4.5933 | 0.0251039 |
| Tfcp2l1 | -1.77041 | 0.0251039 |
| Zfp945 | -1.17178 | 0.0251039 |
| Ecel1 | -3.31826 | 0.0294905 |
| Ppp1r1b | -0.848755 | 0.0294905 |
| Tifa | -1.19657 | 0.0294905 |
| Vip | -3.21695 | 0.0294905 |
| 1110032F04Rik | -1.9602 | 0.0335808 |
| Dtl | -3.70651 | 0.0335808 |
| Pnma2 | -1.02768 | 0.0335808 |
| 3632451O06Rik | 1.66359 | 0.037732 |
| Arhgef2 | 2.33511 | 0.037732 |
| Rasgrp2 | -1.07807 | 0.037732 |
| Rgs9 | -3.84607 | 0.037732 |
| Adcyap1 | -1.9422 | 0.0412 |
| Dbn1 | -2.00756 | 0.0412 |
| Fbxl16 | -0.715843 | 0.0412 |
| Gm5083 | 1.04339 | 0.0412 |
| H2A | -5.33527 | 0.0412 |
| Nrarp | -1.11059 | 0.0412 |
| Pmepa1 | -0.720206 | 0.0412 |
| Elfn2 | -1.08351 | 0.048762 |
| Gm8801 | 4.81941 | 0.048762 |
| Kcne2 | 1.12433 | 0.048762 |
| Lamc2 | 1.16693 | 0.048762 |
| Myoc | -1.59744 | 0.048762 |
| Oprk1 | -2.36585 | 0.048762 |
| Prkar2b | -0.81374 | 0.048762 |
|  |  |  |

**Table D.** Genes differentially expressed (FDR-adjusted P-value < 0.05) between mice in Blocked and Free activity environments.

| **Gene Symbol** | **Log2 (Blocked /Free activity environment)** | **FDR-adjusted P-value** |
| --- | --- | --- |
| Zfp831 | -5.30167 | 0.007313 |
| Zfp365 | -1.26731 | 0.007313 |
| Vstm2a | -0.839738 | 0.007313 |
| Tmem179 | -1.32087 | 0.007313 |
| Thbs4 | -2.80081 | 0.007313 |
| Tbr1 | -4.18002 | 0.007313 |
| Syt16 | -1.99673 | 0.007313 |
| Synpo | -2.22739 | 0.007313 |
| Syndig1l | -1.28772 | 0.007313 |
| Sstr4 | -2.69705 | 0.007313 |
| Sst | -3.24551 | 0.007313 |
| Sp9 | -5.23464 | 0.007313 |
| Slit1 | -2.5715 | 0.007313 |
| Slc35d3 | -2.93163 | 0.007313 |
| Sh3rf3 | -1.61361 | 0.007313 |
| Scn3a | -1.95204 | 0.007313 |
| Rxrg | -4.66121 | 0.007313 |
| Rtn4rl2 | -3.9803 | 0.007313 |
| Rprml | -3.39604 | 0.007313 |
| Rprm | -2.30715 | 0.007313 |
| Rin1 | -2.43658 | 0.007313 |
| Rgs4 | -2.01424 | 0.007313 |
| Rcn1 | -1.09949 | 0.007313 |
| Rasgrf2 | -1.69552 | 0.007313 |
| Rab40b | -2.79305 | 0.007313 |
| Ptprv | -3.59163 | 0.007313 |
| Prss12 | -3.45094 | 0.007313 |
| Prr7 | -2.10579 | 0.007313 |
| Prkch | -1.57485 | 0.007313 |
| Prkar2b | -1.08275 | 0.007313 |
| Pnma2 | -1.15103 | 0.007313 |
| Plk2 | -1.14495 | 0.007313 |
| Pde10a | -3.05835 | 0.007313 |
| Pak6 | -3.34825 | 0.007313 |
| Nts | -3.26483 | 0.007313 |
| Nsg2 | -0.865573 | 0.007313 |
| Npas2 | -1.60414 | 0.007313 |
| Nov | -1.62828 | 0.007313 |
| Nkain2 | -0.946799 | 0.007313 |
| Ngef | -2.74494 | 0.007313 |
| Neurl1b | -3.50048 | 0.007313 |
| Neto1 | -2.88871 | 0.007313 |
| Myo5b | -2.41512 | 0.007313 |
| Mpped1 | -2.67888 | 0.007313 |
| Mkl2 | -0.984511 | 0.007313 |
| Meis2 | -3.01728 | 0.007313 |
| Meis2 | -2.82568 | 0.007313 |
| Mchr1 | -3.23157 | 0.007313 |
| Mal2 | -2.46326 | 0.007313 |
| Lrrc10b | -4.81634 | 0.007313 |
| Lrfn2 | -2.07035 | 0.007313 |
| Lipg | -3.08539 | 0.007313 |
| Lamp5 | -3.57371 | 0.007313 |
| Krt9 | -4.95871 | 0.007313 |
| Klf16 | -1.05624 | 0.007313 |
| Kcnv1 | -5.66069 | 0.007313 |
| Kcnj4 | -5.10231 | 0.007313 |
| Kcnh4 | -3.69834 | 0.007313 |
| Kcnh3 | -2.2069 | 0.007313 |
| Kcng1 | -4.56038 | 0.007313 |
| Kcnf1 | -4.77571 | 0.007313 |
| Kcna4 | -2.04557 | 0.007313 |
| Htr1a | -2.79611 | 0.007313 |
| Grem2 | -1.83284 | 0.007313 |
| Grasp | -1.79957 | 0.007313 |
| Gpr6 | -5.23178 | 0.007313 |
| Gng7 | -2.69011 | 0.007313 |
| Gng4 | -2.53511 | 0.007313 |
| Gda | -5.03023 | 0.007313 |
| Foxg1 | -6.58338 | 0.007313 |
| Fibcd1 | -2.35267 | 0.007313 |
| Fam163b | -1.74646 | 0.007313 |
| Epha4 | -0.851908 | 0.007313 |
| Enc1 | -2.71709 | 0.007313 |
| Egr1 | -1.25297 | 0.007313 |
| Drd1a | -5.47347 | 0.007313 |
| Dlx1 | -6.3096 | 0.007313 |
| Dbpht2 | -2.6186 | 0.007313 |
| D430019H16Rik | -3.33037 | 0.007313 |
| Crhbp | -4.02149 | 0.007313 |
| Creg2 | -1.84155 | 0.007313 |
| Cpne6 | -1.68043 | 0.007313 |
| Cpne5 | -4.77704 | 0.007313 |
| Col6a1 | -1.44883 | 0.007313 |
| Cntnap3 | -4.17254 | 0.007313 |
| Cnih3 | -2.13031 | 0.007313 |
| Chsy3 | -2.23667 | 0.007313 |
| Chst1 | -1.09722 | 0.007313 |
| Chrm3 | -2.07635 | 0.007313 |
| Chrm1 | -5.08345 | 0.007313 |
| Chn1 | -0.984271 | 0.007313 |
| Cdh9 | -2.36943 | 0.007313 |
| Camk2a | -2.26981 | 0.007313 |
| Cacna1h | -2.2123 | 0.007313 |
| Basp1 | -2.22497 | 0.007313 |
| Asic4 | -2.80407 | 0.007313 |
| Ankrd63 | -5.08332 | 0.007313 |
| Alcam | -0.694055 | 0.007313 |
| Akap5 | -2.4136 | 0.007313 |
| AK083197 | -0.898628 | 0.007313 |
| AK047676 | -2.06112 | 0.007313 |
| Adora2a | -4.15213 | 0.007313 |
| Adcy5 | -1.62925 | 0.007313 |
| Actn2 | -3.15911 | 0.007313 |
| 6330403A02Rik | -1.9617 | 0.007313 |
| 3110035E14Rik | -1.86107 | 0.007313 |
| 2010300C02Rik | -1.6219 | 0.007313 |
| 1700086L19Rik | -2.20421 | 0.007313 |
| Vip | -3.45273 | 0.013303 |
| Sgsm2 | -0.685502 | 0.013303 |
| Nrarp | -1.13431 | 0.013303 |
| Kcnj2 | -1.11577 | 0.013303 |
| Kcnb1 | -0.895102 | 0.013303 |
| Htr2a | -1.68809 | 0.013303 |
| Ecel1 | -3.61078 | 0.013303 |
| Dlx2 | -5.25864 | 0.013303 |
| Cyp46a1 | -0.813245 | 0.013303 |
| Crh | -3.0281 | 0.013303 |
| Begain | -0.848632 | 0.013303 |
| Agap2 | -0.823907 | 0.013303 |
| Prss56 | -5.70804 | 0.018063 |
| Ppp1r1b | -0.995275 | 0.018063 |
| Htr1f | -2.97895 | 0.018063 |
| Galnt14 | -1.57624 | 0.018063 |
| Elfn2 | -1.12397 | 0.018063 |
| Ddn | -6.56249 | 0.018063 |
| Ddit4l | -1.56877 | 0.018063 |
| Dbn1 | -2.20525 | 0.018063 |
| 4930539E08Rik | -2.58978 | 0.018063 |
| 1110032F04Rik | -2.05292 | 0.018063 |
| Oprk1 | -2.64282 | 0.023184 |
| Nrsn1 | -0.712158 | 0.023184 |
| Rgs9 | -4.07408 | 0.028391 |
| Rgs9 | -2.96821 | 0.028391 |
| E130012A19Rik | -1.11256 | 0.028391 |
| Rap2b | -0.819448 | 0.032997 |
| Pcdh8 | -1.79691 | 0.032997 |
| C130074G19Rik | -0.909871 | 0.032997 |
| Timp2 | -0.651252 | 0.037753 |
| Sox11 | -1.01575 | 0.037753 |
| Pcdh10 | -0.905815 | 0.037753 |
| Kctd16 | -1.92772 | 0.037753 |
| Ly6h | -1.26516 | 0.042815 |
| Tfcp2l1 | -1.66922 | 0.046735 |
| Rgs2 | -0.726233 | 0.046735 |
| Kcnt2 | -2.88618 | 0.046735 |
| Dtl | -3.54164 | 0.046735 |
| Astn1 | -0.603449 | 0.046735 |
